# Tracking Extensive Portfolio of Cyanotoxins in Five-Year Lake Survey and Identifying Indicator Metabolites of Cyanobacterial Taxa

**DOI:** 10.1021/acs.est.4c04813

**Published:** 2024-08-30

**Authors:** Xuejian Wang, Simon Wullschleger, Martin Jones, Marta Reyes, Raphael Bossart, Francesco Pomati, Elisabeth M.-L. Janssen

**Affiliations:** † Swiss Federal Institute of Aquatic Science and Technology (EAWAG), Dübendorf 8600, Switzerland; ‡ School of Biosciences, University of Birmingham, Edgbaston, Birmingham B15 2TT, United Kingdom

**Keywords:** microcystin, suspect screening, monitoring, cyanopepetides, harmful algal bloom

## Abstract

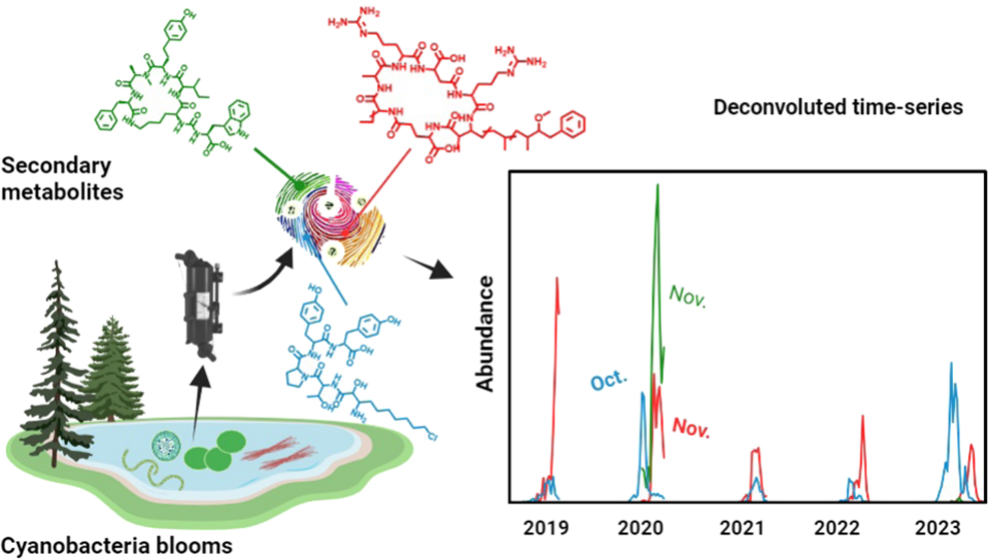

Cyanobacterial blooms require monitoring, as they pose a threat to ecosystems and human health, especially by the release of toxins. Along with widely reported microcystins, cyanobacteria coproduce other bioactive metabolites; however, information about their dynamics in surface waters is sparse. We investigated dynamics across full bloom successions throughout a five-year lake monitoring campaign (Greifensee, Switzerland) spanning 150 sampling dates. We conducted extensive suspect screening of cyanobacterial metabolites using the database CyanoMetDB. Across all 850 samples, 35 metabolites regularly co-occurred. Microcystins were present in 70% of samples, with [d-Asp^3^,(*E*)-Dhb^7^]MC-RR reaching concentrations of 70 ng/L. Anabaenopeptins, meanwhile, were detected in 95% of all samples with concentrations of Oscillamide Y up to 100-fold higher than microcystins. Based on LC-MS response and frequency, we identified indicator metabolites exclusively produced by one of three cyanobacteria isolated from the lake, these being [d-Asp^3^,(*E*)-Dhb^7^]MC-RR from *Planktothrix* sp. G2020, Microginin 761B from *Microcystis* sp. G2011, and Ferintoic acid B from *Microcystis* sp. G2020. These indicators showed distinct temporal trends and peaking seasons that reflect the variance in either the abundance of the producing cyanobacteria or their toxin production dynamics. Our approach demonstrates that selecting high LC-MS response and frequent and species-specific indicator metabolites can be advantageous for cyanobacterial monitoring.

## Abstract 2

This work provides toxin and cyanopeptide profiles across a 5-year lake campaign with new insights from selecting frequent and species-specific indicator metabolites that allow deconvoluting blooms of co-occurring cyanobacterial taxa.

## Introduction

Harmful cyanobacterial blooms (HCBs), also referred to as harmful algal blooms (HABs), are increasing in frequency, magnitude, and duration across the globe.^1^ Their occurrence can pose significant risks to both human and ecosystem health, in particular, due to the release of toxic secondary metabolites, i.e., cyanotoxins from constituent cyanobacterial species.^2,3^ Recognizing the significant risks posed by some of these metabolites, the World Health Organization (WHO) has set recommended threshold concentrations in water bodies used for drinking water or for recreational activities for four cyanotoxins, hereafter referred to as “WHO toxins,” namely, Microcystin-LR (MC-LR), anatoxin-a, saxitoxin, and cylindrospermopsin.^4^ MC-LR is the most frequently reported of these cyanotoxins; it causes liver and kidney damage,^5^ and has even been linked to human death, following acute exposure.^6^ A wealth of studies have monitored the abundance of cyanobacteria^7−11^ and WHO toxins in lakes and explored their correlations with other environmental parameters (e.g., temperature, nutrients).^12−16^

Besides the widely known WHO toxins, many more bioactive secondary metabolites are coproduced by cyanobacteria.^11,17,18^ Approximately 65% of known cyanobacterial metabolites are peptide-based compounds, i.e., cyanopeptides. These are grouped into different classes based on similarities in their chemical structures, for example, microcystins, anabaenopeptins, microginins, or cyanopeptolins (some structures are presented in Table 1).^19,20^ Cyanopeptides belonging to these classes have been repeatedly shown to adversely affect aquatic invertebrates.^11,21−26^ For example, anabaenopeptins have been shown to inhibit phosphatases and carboxypeptidases,^27,28^ cyanopeptolins are potent inhibitors of serine proteases,^15^ and microginins inhibit zinc metalloproteases.^15,29^ Exploration of the co-occurrence of these cyanobacterial metabolites could enhance the understanding of relevant exposure to mixtures that need to be considered for risk assessment.

**Table 1:**
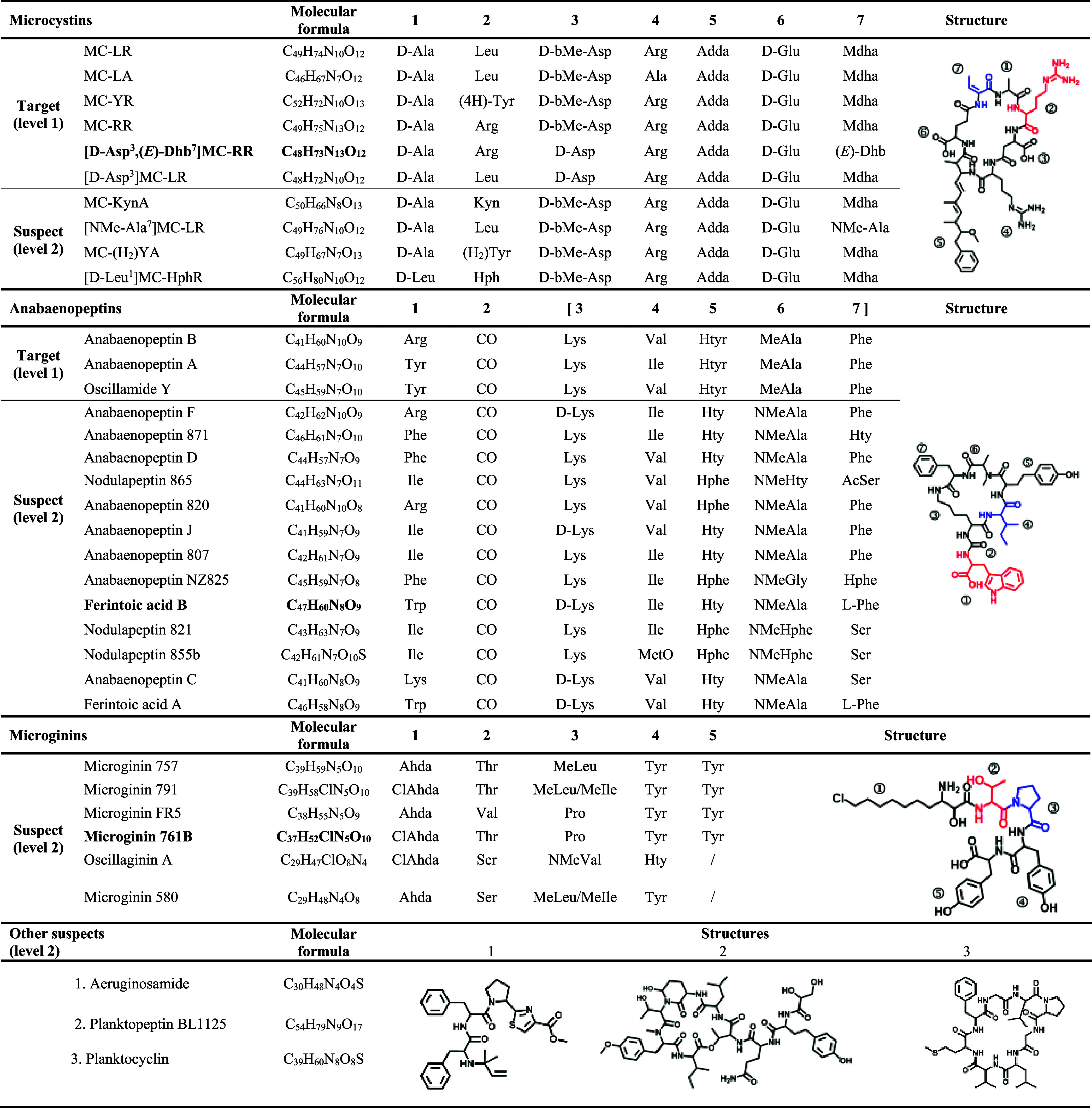
Cyanobacterial Metabolites Identified in Lake Greifensee Samples from the 2019-2023 Lake Monitoring Campaign^a^

a The blue and red colors in metabolite structures indicate the positions of the most variable building blocks, and the corresponding compound names for structures shown on the right are indicated in bold font.

Besides toxicological aspects and exposure to mixtures, frequent and abundant metabolites that co-occur with toxic cyanobacteria strains and/or with WHO toxins can aid monitoring of cyanobacterial blooms. Cyanopeptides other than microcystins can occur with similar frequency and at comparable concentrations to microcstins in surface waters.^11,30−32^ Recent studies covered a wider range of cyanobacterial secondary metabolites and explored their correlation with cyanobacteria and nutrients.^33,34^ Miller et al. present one of few studies that enabled investigation of the temporal variability of 8 microcystins, 3 anabaenopeptins, and 3 cyanopeptolins based on automatic sampling every 6 h during a two-month blooming season in Lake Winnebago.^15^ Their results suggest that variability in toxin profiles was strongly correlated with time and the C/N ratio of the toxin pool.^15^ Another six-year lake study reported increased duration of cyanobacterial blooms and a negative correlation between anabaenopeptin and mirocystin abundance linked to zebra mussel invasion.^16^ The emergence and disappearance of different chemotypes, i.e., oligopeptide patterns, can at times also be linked to the variable abundance of specific cyanobacteria taxa, as reported for *Planktothrix*.^13^

Studies with high-frequency sampling across multiple years are essential to better understand the variability of harmful cyanobacterial blooms. Metabolites that are produced exclusively by specific cyanobacteria taxa may serve as indicators of the occurrence of that taxa and their variable abundance thereof, as well as their productivity in the lake. Yet, the temporal dynamics of most cyanopeptides in lakes, besides microcystins, are limited. Especially long-term monitoring data covering multiple seasons, including the pre- and postbloom periods, is required for interseasonal and multiannual time comparisons.

In this study, we tracked a comprehensive portfolio of cyanobacterial toxins and secondary metabolites across a five-year lake campaign at Lake Greifensee, Switzerland. We demonstrate how a legion of other metabolites consistently co-occurred with microcystins and how coproduction dynamics differ across seasons. We further analyzed the metabolite profiles of three cyanobacteria strains isolated from the lake during bloom events and compared those to their *in situ* occurrence. We identified indicator metabolites of specific cyanobacterial taxa that show distinct temporal trends and peaking seasons in Lake Greifensee. Overall, selection of high LC-MS response and frequent and species-specific indicator metabolites can be advantageous for monitoring cyanobacterial blooms.

## Materials and Methods

### Study Site, Sampling, and Sample Preparation

Greifensee is a perialpine lake located in northeastern Switzerland, with a surface area of 8.45 km^2^ and a maximum depth of 33 m. The lake has a history of eutrophication and reoligotrophication,^35^ is currently meso-eutrophic,^36^ and completely mixes once in the winter.

Samples were collected at a monitoring platform (47.36640°N, 8.66511°E) situated toward the outlet of the lake, at which the lake is 20 m deep. Weekly samples were taken during the following time periods: May to November 2019, March to November 2020, April to November 2021, April to November 2022, and March to December 2023, spanning a total of 150 sampling dates. Samples were collected at 3 m depth, the middle of the photic zone, where the epilimnion is well mixed in Lake Greifensee. In addition, a phytoplankton monitoring system is located at this sampling location and depth, providing information about community composition. Samples were collected with a 5L Niskin bottle (Hydrobios) and filtered in the field through a 100 μm metal mesh by gravity to remove large particulate matter and zooplankton. Visual inspection in the field verified that no larger phytoplankton aggregates were removed by this prefiltration, though we cannot exclude the possibility that a minor portion of biomass was retained. Three replicate Niskin pulls were carried out per sampling event, with 2 L of each pull being stored in glass bottles, in the dark, inside of a cooler box for transport to the laboratory.

In the laboratory, the native pH of each 2 L sample was noted and adjusted to pH 9 by using 1 M NaOH and 1 M HCl. Next, samples were filtered through a glass fiber filter (47 mm, 0.7 μm, Whatman, prerinsed with 100 mL of nanopure water) using a vacuum pump (KNF, Germany). The glass fiber filters used to process each 2 L replicate were transferred into a 15 mL Falcon tube (referred to as “biomass sample”), while 40 mL of corresponding filtrate, hereafter referred to as “aqueous sample,” was transferred into a 50 mL Falcon tube. Filters and filtrate were both stored at −20 °C. The frozen filters were lyophilized (Christ α 2–4 LSCplus) for 8 h at 0.04 mbar and stored at −20 °C. Filters were extracted by adding 10 mL of 70:30% v/v methanol/nanopure water solution and vortex mixing (Vortex Genie 2) for 10 s, followed by agitation in an ultrasonic water bath (VWR Ultrasonic Cleaner USC-THD) operated at 40 °C and at maximum power for 30 min. Biomass extracts were then centrifuged for 10 min at 4000 *g* at room temperature (Heraeus Megafuge 1.0), and 8 mL of the extract was transferred into a glass vial. This extraction procedure was repeated, and the resulting extract was combined with the from the first round of extraction. The 16 mL extract was concentrated at 40 °C under a gentle stream of nitrogen gas (0.5–3.0 L/min over a 2 h ramp) using a Turbovap LV (Biotage, Sweden) to remove the majority of the methanol. After the evaporation, when approximately 30% of the initial volume remained, the solution was transferred to a new glass vial and gravimetrically adjusted with nanopure water to approximately 5 g (exact masses were noted). These biomass extracts were stored at −20 °C. Biomass extracts and aqueous samples were freshly thawed and centrifuged for 10 min at 4000 *g* at room temperature (Heraeus Megafuge 1.0) and diluted with nanopure water (10 to 500-fold) before analysis.

### Analysis of Toxins and Secondary Metabolites

Samples were thawed and analyzed within 24 h. All samples from 1 year were analyzed in 2 batches, one for the aqueous samples and one for the biomass extracts, and each batch was analyzed in one sequence on the analytical instrument, with 10 sequences in total. For each sequence, quality control samples (standard mixture of known metabolites) were injected at least every 24 h to facilitate assessment of the sensitivity and accuracy of the analysis, and blank samples (nanopure water) were used between sets of replicates to avoid carry-over effects. External calibration curves in nanopure water of reference standards and bioreagents were measured at the beginning and end of each sequence, and samples outside of the calibration range were diluted and reinjected. In each sequence, a dilution series of the highest concentrated sample was added at the end to verify that signals decrease linearly with dilution.

The analysis was performed by high-performance liquid chromatography (HPLC; Dionex UltiMate3000 RS pump, Thermo Fisher Scientific) coupled with high-resolution mass spectrometry (HRMS(/MS); Fusion Lumos, Thermo Fisher Scientific). A previously validated method was used that included an automated enrichment and cleanup of samples by online solid phase extraction (online SPE, 20 mg Oasis HLB sorbent, 15 μm).^37^ Briefly, 10 mL of the sample was enriched onto the online SPE system and then washed and eluted with a combination of 30% nanopure water and 70% methanol. The SPE eluate was automatically diluted with water to refocus analytes at the inlet of the analytical HPLC column (Kinetex C_18_, 2.6 μm, 2.1 mm × 100 mm, Phenomenex), operated at 40 °C and fitted with a precolumn (VanGuard Cartridge, Waters). Analytes were separated using a binary gradient of nanopure water (mobile phase A) and methanol (mobile phase B), both containing 0.1% v/v formic acid, supplied at 0.255 mL/min, as follows: 20.04/28.63/50.04/70.02/100/100/20.04/20.04% B at 0/12/16/32/32.1/37/37.1/42.1 min. Analytes eluted from the analytical column underwent positive-mode electrospray ionization, using a heated electrospray ionization (H-ESI) source: 320 °C capillary temperature, 3.5 kV electrospray voltage, 40 arbitrary units (AU) of sheath gas, 10 AU of auxiliary gas and 0 AU of sweep gas, S-lens 40%RF. Full-scan data were acquired between 450–1350 *m*/*z* at 120 000 resolution (full width half-maximum at 200 *m*/*z*; fwhm_200 *m*/*z*_) in profile mode, using 1 × 10^5^ auxiliary gain control (AGC) target, 50 ms maximum ion injection time, 1 microscan, and wide quadrupole isolation. Tandem mass spectrometry data were acquired at 15000 resolution (fwhm_200 *m*/*z*_) in centroid mode for the top-3 ions from the preceding full scan. Higher-energy collisional dissociation (HCD) MS^2^ spectra were acquired consecutively, at normalized collision energies of 15, 30, and 45%, with CyanoMetDB as the inclusion list^37,38^ and 5 s dynamic exclusion time.

Data analysis and peak area extraction were performed with Skyline 20.1 (MacCoss Lab Software), as previously reported.^39^ Targeted analysis was performed for compounds where a reference standard or bioreagent was available (SI-1: Text S1 and Table S1), and criteria for identification were based on exact mass (<5 ppm mass error), accurate isotopic pattern of the precursor ion (Skyline idotP > 0.95 considering the top three isotopes), and match of retention time and MS^2^ spectra of reference materials (SI-2). The peak area of the protonated precursor ion was extracted and quantified by using external calibration curves of the target cyanopeptides in nanopore water. Matrix-matched calibration curves using a mix of samples of each sequence demonstrated that effects were comparable, as previously reported.^37^ The concentrations in the online SPE sample vial were corrected for each sample individually based on the dilution of the extract before LC-MS/MS analysis (10 to 500-fold), the total volume of the extract (as noted for each sample, approximately 5 g), and the volume of lake water filtered (1–2 L depending on the density of the biomass).

In addition, suspect screening analysis was conducted for all compounds for which no reference material was available. First, full-scan (MS^1^) spectra were screened for exact masses (<5 ppm mass error) and accurate isotopic patterns of the precursor ion (Skyline idotp >0.95) for metabolites reported in the cyanobacterial suspect list CyanoMetDB.^20,38^ Then, MS^2^ spectra corresponding to tentative candidates were manually annotated and supported where possible, by in-silico fragmentation predictions (Met Frag Web with CyanoMetDB Version02 2023 database^38^). Predicted and measured spectra were manually evaluated and the level of confidence assessed.^40^ Metabolites were classified as a tentative candidate (Level 3) based on exact mass (<5 ppm mass error), accurate isotopic pattern (Skyline idotp value >0.9), and evidence from fragmentation data; a probable structure (Level 2) based on additional, comprehensive MS^2^ fragmentation information that helped to confirm the connectivity of molecular substructures; or a confirmed structure (Level 1) when these parameters were in agreement with available reference standards or bioreagents (SI-1: Table S1). The peak areas of selected ion chromatograms were extracted for all metabolites classified as level 1 or 2 annotations.

### Cyanobacteria Isolates from Lake Greifensee

Three cyanobacteria strains were isolated from Lake Greifensee during bloom events. *Microcystis* sp., named G2011, was isolated in 2011 and shows 100% similarity to *M. aeruginosa* (16S RNA region Basic Local Alignment Search Tool, BLAST result SI-1: Table S2) confirmed by microscopy identification. Polymerase chain reaction (PCR) analysis revealed the presence of microcystin synthetase A (*mcyA*) gene and *mcyE* gene after isolation.^41^ However, a recent PCR analysis in 2022 of the isolated culture showed a negative response for the two genes, suggesting the possible loss of microcystin production capacity (SI-1: Table S2). The other two cyanobacteria strains were isolated in 2020, by picking single-cell colonies. The initial medium for culturing was 50% filtered lake water (0.2 μm cellulose filter) and 50% WC medium (SI-1: Text S1) and later transferred to pure WC medium.^42^ Microscopy identification suggested that one strain was *Planktothrix* sp. named G2020, with 100% similarity to *P. agardhii* and 99.84% similarity to *P. rubescens*. The other isolate from 2020 was another *Microcystis* sp. named G2020, with 99.84% similarity to *M. aeruginosa* (SI-1: Table S2 and Figure S1). PCR analysis suggested that *Planktothrix* G2020 was positive for a *mcyE* gene, while *Microcystis* G2020 was negative for *mcyE*. All three strains tested negative for the *anaC* gene, part of the anatoxin-a synthetase gene cluster. The biomass extracts from laboratory cultures of the three isolated strains were analyzed for cyanobacterial metabolites using the HPLC-HRMS/MS method described above.

### Statistical Analysis

Correlations among cyanobacterial metabolites in Lake Greifensee were explored, using Spearman’s rank correlation coefficient (ρ) with R studio (version 4.3.0), to define metabolites with similar variance patterns across the five-year sampling campaign. The study applied Spearman’s rank correlation coefficient since it does not require specific assumptions and provides consistent results with nonlinear relationships.^14^

## Results and Discussion

### Diversity of Metabolites

The five-year lake monitoring campaign at Greifensee between 2019 and 2023 included 150 sampling dates with a total of 859 samples (449 aqueous phase and 410 biomass extracts) that were analyzed for cyanobacterial secondary metabolites using targeted and suspect screening approaches. Overall, 46 different cyanobacterial metabolites were detected based on comparison with the database CyanoMetDB. Of these, 6 microcystins and 3 anabaenopeptins were identified with reference to chemical standards or bioreagents, yielding the highest confidence identifications (Level 1); hereafter, we refer to these as “targeted metabolites.” The remaining 37 metabolites were annotated by suspect screening, with confidence in annotation quality ranging from level 3 “tentative candidates” (11 metabolites, SI-1: Table S3) to level 2 “probable structures” (26 metabolites), where the latter is based on comprehensive interpretation of their MS^2^ spectra (SI-2). For further analysis, we focused on 35 metabolites, i.e., targeted metabolites and metabolites annotated to confidence level 2, which belong to three compound classes: microcystins (*n* = 10), anabaenopeptins (*n* = 16), and microginins (*n* = 6), in addition to aeruginosamide, Planktopeptin BL1125, and Planktocyclin (Table 1).

### Targeted Metabolites

**Figure 1:**
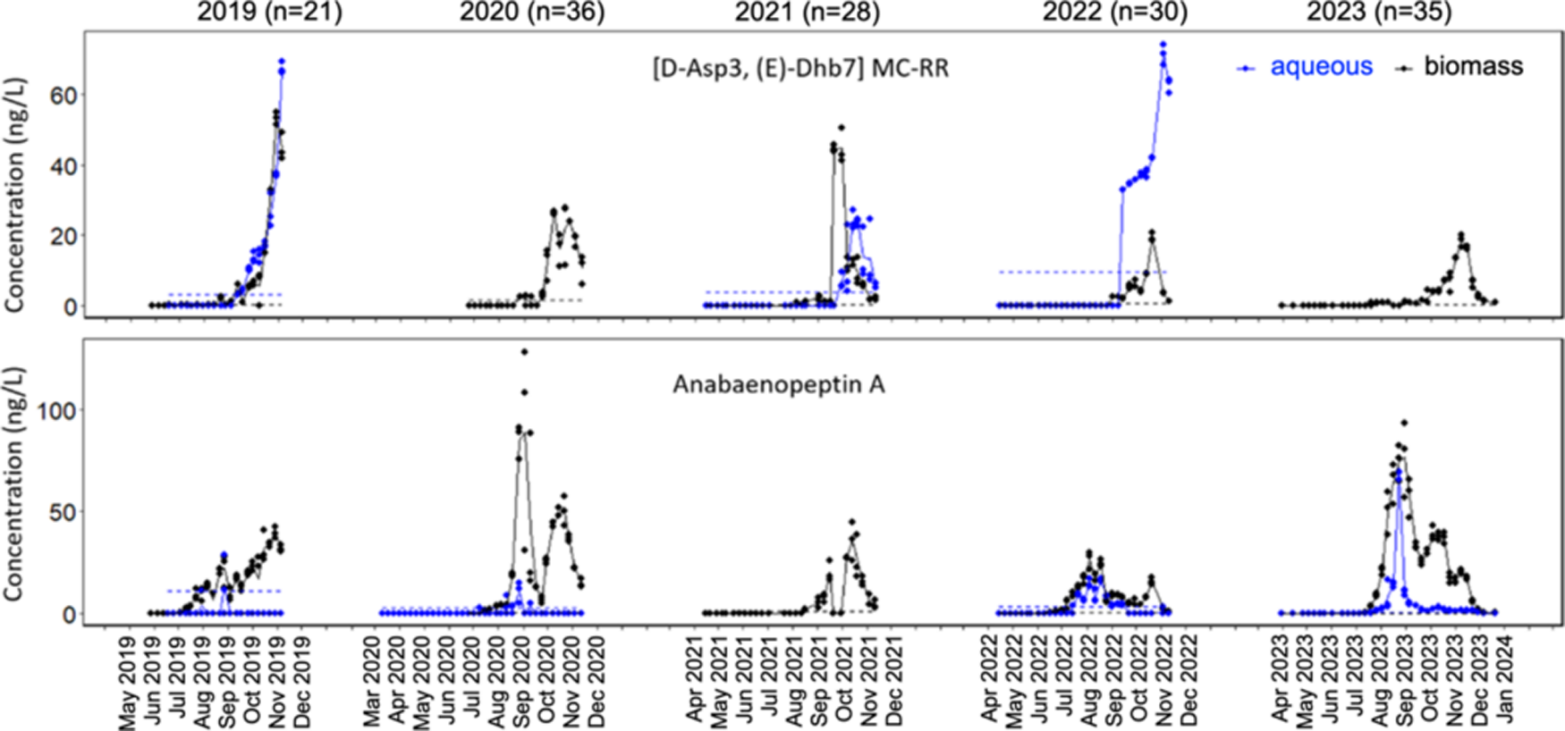
Concentrations in ng/L of [d-Asp^3^,(*E*)-Dhb^7^]MC-RR (top) and Anabaenopeptin A (bottom) in Lake Greifensee from 2019 to 2023, with *n* = 21–36 annual sampling dates for biomass (black, triplicates) and aqueous samples (blue, triplicates). Dashed lines indicate the limit of quantification for each annual data set run on separate LC-MS sequences each year. Each data point represents one sample out of triplicate samples collected on each sampling date. Solid lines connect the average concentration of these triplicate samples on each sampling date, while white spaces between data sets reflect the dates where no sampling took place.

For the 9 targeted metabolites, we obtained absolute concentrations, and the time series are shown for the most dominant microcystin, [d-Asp^3^, (*E*)-Dhb^7^]MC-RR, and Anabaenopeptin A in Figure 1. For [d-Asp^3^, (*E*)-Dhb^7^]MC-RR, concentrations in biomass and aqueous samples generally peaked in late summer and autumn, reaching up to 70 ng/L. Several peaks with lower concentrations were observed, suggesting possible multibloom periods during each year. In 2020, 2021, and 2023, the highest peak concentrations occurred in biomass samples. Note that concentrations are reported as ng/L, as this reflects the amount of metabolite, in ng, quantified in biomass filtered out of 1 L lake water. We recorded the weight of the filters before and after filtration as well as after lyophilizing, to attempt to quantify the biomass of all samples collected in 2019. However, the amount of biomass was too low to return reliable results throughout most of the campaign. Hence, we report concentrations of targeted metabolites in biomass samples as ng/L. The concentrations of [d-Asp^3^, (*E*)-Dhb^7^]MC-RR in aqueous samples from 2019, and especially 2022, were higher than those of corresponding biomass samples. This was not observed for the other target microcystins (SI-1: Figure S2 panels 3–7). In 2019, the sampling campaign concluded in November, and after analysis of the samples, it became apparent that the concentration was on a continuous rise until then. Therefore, in the following years, we extended the sampling period and concluded that sampling until mid-December is necessary to capture the peak concentration.

Oscillamide Y was the most abundant anabaenopeptin with concentrations up to 2000 ng/L, 2 orders of magnitude higher than the most abundant microcystin (SI-1: Figure S2 panel 2). However, the concentration was calculated from a bioreagent with relatively lower purity compared with reference standards. Thus, we show the variance of Anabaenopeptin A in Figure 1, the second most abundant target anabaenopeptin. The highest peak of Anabaenopeptin A was 90 ng/L. In contrast to [d-Asp^3^, (*E*)-Dhb^7^]MC-RR, the years 2020 and 2023 showed the highest concentrations for Anabaenopeptin A in biomass samples in autumn, analogous to the other two target anabaenopeptins; Anabaenopeptin B and Oscillamide Y (SI-1: Figure S2 panel 1–2). Overall, across all 9 targeted metabolites, higher metabolite diversity and concentrations were detected in biomass samples than in aqueous samples (SI-1: Figure S3).

[d-Asp^3^, (*E*)-Dhb^7^]MC-RR was the most abundant microcystin, though its highest concentration was 300-fold lower than the WHO MC-LR guideline value for recreational activities (24 μg/L).^4^ The concentration of MC-LR was even below 5 ng/L at peak concentrations (SI-1: Figure S2 panel 4). While the microcystin concentrations are low compared to those of eutrophic lakes during bloom events, the anabaenopeptin concentrations are rather comparable. For example, a monitoring campaign at Lake Winnebago (Wisconsin, 68.75 days in 2013) reported peak concentrations of MC-LR of 10 μg/L, more than 1000-fold higher than the concentration of MC-LR in Lake Greifensee.^15^ In contrast, the study reported that in the same samples, the median Anabaenopeptin A and Anabaenopeptin B concentrations were both in the range of 0.1 to 1 μg/L, consistent with observations in Lake Greifensee. Another study at Lake Mendota (Wisconsin, 2013–2019) reported consistently higher anabaenopeptin concentrations compared to co-occurring microcystins, in line with the composition in Lake Greifensee.^16^ Concentrations of microcystins and anabaenopeptins were comparable in Lake Zürichsee and at times higher for microcystins in Lake Hallwilersee, two other Swiss lakes,^37^ highlighting that the ratio of these metabolite classes can be variable not only temporally within one lake but also across lakes.

### Suspect Metabolites

Besides the 9 identified targeted metabolites, 26 additional metabolites were identified with level 2 confidence (tentative structures) based on suspect screening and comprehensive MS^2^ annotation. As no reference standards or bioreagents were available for these suspects, their absolute concentrations could not be determined. Instead, we explored the relative concentration changes of these metabolites based on raw peak area values. The time course of peak areas across the 5-year sampling campaign for each suspect metabolite is shown in Figure S4 (SI-1). These suspect metabolites generally show highest abundance in summer (July–August) or autumn (September–November). We further analyzed the entire targeted and suspect metabolite data set to identify which of these metabolites had high LC-MS response (i.e., peak area) and occurred frequently, and thus prove useful as potential indicator metabolites for future bloom monitoring.

### Detectability and Frequency

**Figure 2:**
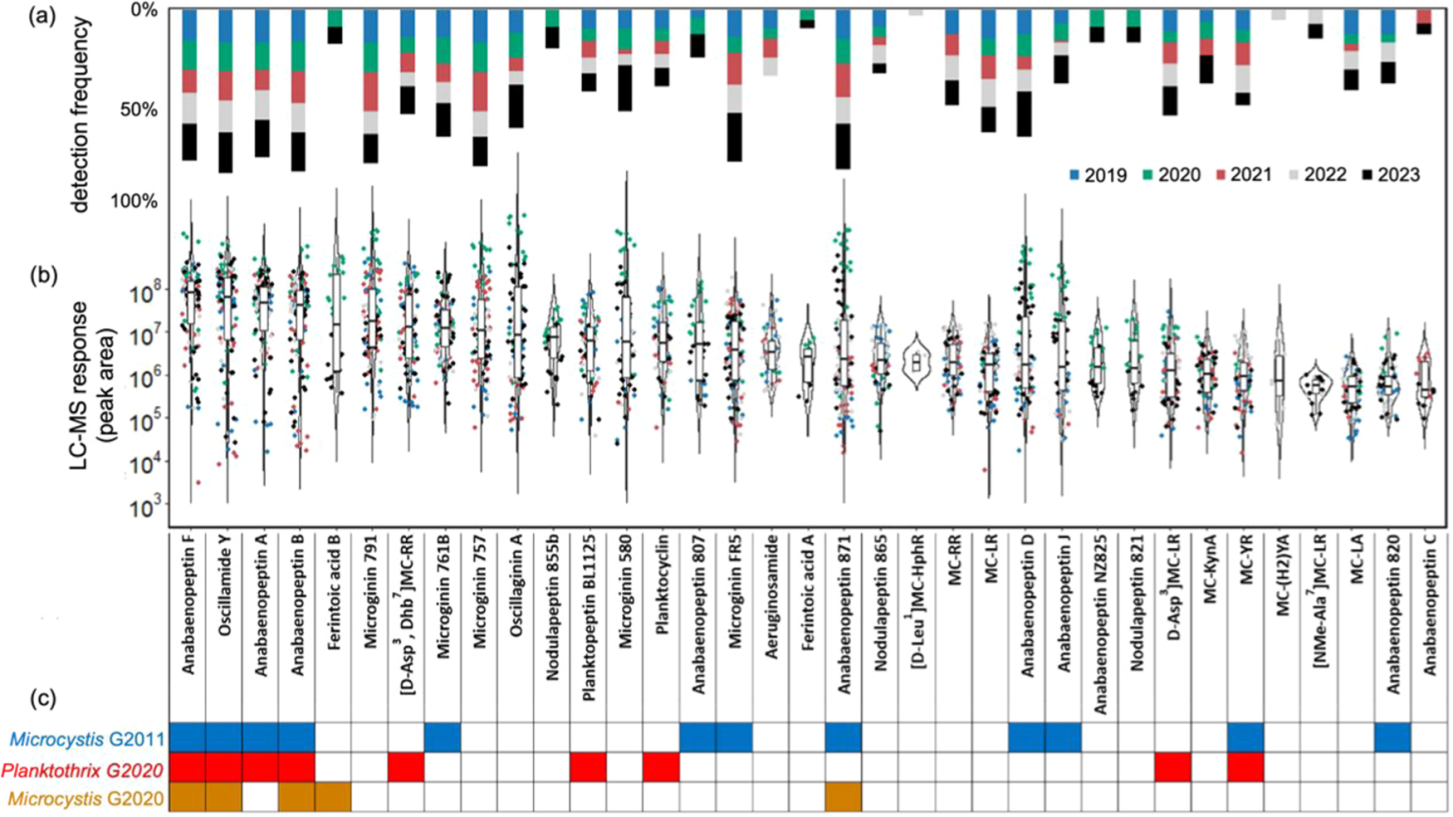
(a) Relative detection frequency and (b) LC-MS response (peak area) for 35 metabolites in Lake Greifensee over a 5-year sampling campaign spanning from 2019 to 2023 (color-coded), and (c) presence of representative metabolites in cyanobacteria isolated from Lake Greifensee, *Microcystis* G2011 (blue), *Planktothrix* G2020 (red), and *Micorcystis* G2020 (yellow ocher).

In this study, the absolute concentration of 26 suspect metabolites could not be calculated due to a lack of available reference standards. We therefore focus on the LC-MS response of each metabolite, i.e., their peak area values, to evaluate the detectability of these metabolites. By comparing peak area values, we aimed to highlight those metabolites that are likely to be detected or detectable in the future by LC-MS-based monitoring work. Data in Figure 2b summarize the peak area range for all identified (i.e., targeted) and suspected (level 2 annotation) metabolites detected as part of the five-year sampling campaign in Lake Greifensee, ranked in descending order of median values (results for aqueous samples in SI-1: Figure S5). Peak area values spanned 6 orders of magnitude. [d-Asp^3^, (*E*)-Dhb^7^]MC-RR and Anabaenopeptin A were ranked seventh and third, respectively, among the 35 metabolites. Among the top 10 metabolites with consistently highest median peak area values, the first five places are taken by anabaenopeptins (Anabaenopeptin F, Oscillamide Y, Anabaenopeptin A, Anabaenopeptin B, Ferintoic acid B), four are microginins (Microginin 791, Microginin 761B, Microginin 757, and Oscillaginin A), and one is a microcystin, namely, [d-Asp^3^, (*E*)-Dhb^7^]MC-RR.

In addition to most responses, i.e., detectable metabolites, we also focused on the frequency with which metabolites were detected as a way to further prioritize metabolites for future monitoring work. Detection frequency was calculated by dividing the number of dates at which the metabolite was detected by the total number of sampling dates across the campaign. The detection frequencies for each metabolite in biomass samples are shown in Figure 2a (results for aqueous samples are given in SI-1: Figure S6). The colors for the bar chart align with the color of the data points in the violin plot, indicating different frequencies in each year across the whole sampling period. Most metabolites (n = 22) were detected in all 5 years and appeared with comparable frequencies in each year. For example, Anabaenopeptin F, the metabolite with the highest LC-MS response based on peak area values, was detected in 78% of all samples with an equal annual frequency across the five-year campaign.

Several metabolites with high LC-MS response (left side of Figure 2) also showed high detection frequency, e.g., Anabaenopeptin F, Oscillamide Y, Anabaenopeptin A, Anabaenopeptin B, Microginin 791, and [d-Asp^3^, (*E*)-Dhb^7^]MC-RR. Likewise, several metabolites with comparably low peak area values were, nevertheless, detected with high frequency across the campaign, including Anabaenopeptin D and [d-Asp^3^]MC-LR. On the other hand, 9 metabolites were detected less frequently, i.e., in only 1–2 out of 5 years, including Ferintoic acid B and Nodulapeptin 855b in 2020 and 2023, with relatively high LC-MS response in both years. These less frequent yet high response metabolites suggest that their detection was related to specific conditions of the blooming years rather than the fact that their rare occurrence was related to the detection limit of the LC-MS method. Comparing the detection frequency of each metabolite class demonstrates that microcystins were detected on 70% of the sampling dates, while anabaenopeptins and microginins were detected more frequently with 95% and 94%, respectively (SI-1: Table S4). Combining the detection frequency and LC-MS response data (peak area), we conclude that microcystins frequently occurred in biomass samples but anabaenopeptins and microginins dominated the metabolite profile between 2019 and 2023 in Lake Greifensee.

### Pigments and Indicator Metabolites

Besides focusing on cyanobacterial metabolites in the lake, environmental parameters were also acquired from the monitoring platform, including Chlorophyll-a (Chl-a), phycocyanin, oxygen saturation, water temperature, pH, total phosphorus, and total nitrogen. Chl-a is often used as an indicator for cyanobacteria and included in the alert level framework by the WHO, provided that the chlorophyll stems largely from cyanobacteria.^4^ However, in the presented study, we did not observe a consistent correlation between cyanobacterial metabolite patterns and Chl-a or any other monitoring parameter (Spearman’s rank coefficients, ρ values range from −0.55 to 0.33, SI-1: Table S5, Figures S7–S9). For example, in August–September 2019, Oscillamide Y and Chl-a showed a similar peaking trend, but thereafter, the concentration of Oscillamide Y increased again while the concentration of Chl-a kept decreasing (Figure S8). Monitoring of the phytoplankton (SI-1: Figure S10) confirmed that green algae and other taxa coexist and contribute to Chl-a signal.^43,44^ Thus, Chl-a is not always a reliable indicator parameter for cyanobacteria or the presence of their toxic metabolites in Lake Greifensee. Besides Chl-a, phycocyanin data were available from July 2020 to December 2023. Similar to Chl-a, no systematic correlation between phycocyanin and cyanobacterial metabolites was observed (Spearman’s rank coefficients, ρ values range from 0.14 to 0.54, SI-1: Table S5, Figures S7–S9). The phycocyanin to Chl-a ratio, which reduces errors due to pigment quenching effects, shows, in general, better, yet still inconsistent, correlations with toxin patterns (Spearman’s rank coefficients, ρ values range from 0.17 to 0.62, SI-1: Table S5, Figure S7). For example, the Spearman’s rank coefficients for [d-Asp^3^, (*E*)-Dhb^7^]MC-RR and Oscillamide Y with Chl-a, phycocyanin, and phycocyanin to Chl-a ratio increased from −0.05 to 0.31 and to 0.54 and from −0.09 to 0.31 and to 0.54, respectively (SI-1, Table S5). As these traditional monitoring parameters were not reliable indicators of toxic level in the lake, we further evaluated whether specific metabolites can serve as indicators for the presence of cyanobacteria and individual taxa.

A variety of cyanobacterial metabolites were detected in Lake Greifensee, and they are likely produced by multiple co-occurring cyanobacteria species. A previous study pointed out that a total of 42 cyanobacterial species from 26 genera, mainly of the order *Chroococcales*, *Synechococcales*, *Nostocales*, *Osillatoriales* and *Pseudanabaenales* were identified in pelagic samples from Lake Greifensee, between 1974 and 2010.^45^ Some of these cyanobacterial taxa can contribute to blooms and, hence, dominate the toxin and metabolite profiles. The metabolites specifically produced by each species may reflect a combination of the species abundance and/or the production dynamics of the producing species in the lake. Here, we investigated how the metabolite profile of individual species isolated during prior bloom events in Lake Greifensee might help to guide the selection of specific indicator metabolites for lake monitoring purposes. We acknowledge that changes in cyanobacteria physiological state, e.g., due to laboratory culture conditions differing from environmental conditions, may influence the variety and quantity of secondary metabolites produced. Thus, “indicator metabolites” in this study served as alerting metabolites, suggesting the possible presence of producing strains, the confirmation of which would require further investigations.

Data in Figure 2c show that 16 of the 35 metabolites identified in Lake Greifensee, including the top-5 metabolites based solely on LC-MS peak area values, can be produced by one or more of the three cyanobacterial isolates *Microcystis* G2011, *Planktothrix* G2020, and *Microcystis* G2020. These observations indicate that these cyanobacteria may have significantly contributed to the metabolite profiles observed throughout the five-year monitoring campaign. However, over half of the metabolites detected in the lake could not be identified in any of the three strains, most likely because more species coexist, but perhaps due to changes in their production dynamics under laboratory culturing conditions or due to changes in their chemotypes relative to the point in time at which they were first isolated from the lake. Of the 16 metabolites detected in the isolates, the three most abundant anabaenopeptins, namely, Anabaenopeptin F, Oscillamide Y and Anabaenopeptin B in lake samples, can be produced by all three isolated cyanobacterial strains, and these show a strong positive correlation with each other in Lake Greifensee samples (Spearman’s rank coefficient, ρ>0.9, SI-1: Figure S2 panels 1–2, Figure S4 panel 1, Table S5).

In addition, several metabolites were uniquely produced by isolated cyanobacteria. Microginin 761B, Microginin FR5, Anabaenopeptin 807, Anabaenopeptin D, Anabaenopeptin J, and Anabaenopeptin 820 were only produced by *Microcystis* G2011, and only Anabaenopeptin 807, Anabaenopeptin D and Anabaenopeptin J showed moderate autocorrelation among each other (Spearman’s rank coefficient, ρ > 0.74, SI-1: Table S5). The most abundant microcystins [d-Asp^3^, (*E*)-Dhb^7^]MC-RR and [d-Asp^3^]MC-LR, as well as Planktopeptin BL1125 and Planktocyclin, were exclusively produced by *Planktothrix* G2020 and a strong autocorrelation was observed across all metabolites (Spearman’s rank coefficient, ρ = 0.86–0.90, SI-1: Table S6). The *Microcystis* G2020 was the only isolated species producing Ferintoic acid B.

The metabolite profile of *Planktothrix* G2020 from Lake Greifensee is comparable to those of blooms in other Swiss lakes dominated by *Planktothrix rubescens*. For example, in Lake Hallwilersee, the metabolite profile from a bloom of *P. rubescens* also included [d-Asp^3^, (*E*)-Dhb^7^]MC-RR, Planktocyclin, Anabaenopeptin A, Anabaenopeptin B, Oscillamide Y, and Anabaenopeptin F (or Anabaenopeptin E, isomers not differentiated).^13^ Toxic strains of *M. aeruginosa* are well known for producing microcystins; however, of the two *Microcystis* strains isolated from Lake Greifensee, only G2011 produced MC-YR, and in low quantities.

Through collective consideration of which metabolites are detected frequently in Lake Greifensee samples, with high LC-MS response (peak area) and that have been found to occur in specific isolated cyanobacterial strains from the lake, we propose four indicator metabolites that may allow for monitoring of different cyanobacterial bloom dynamics in Lake Greifensee: Microginin 761B for *Microcystis* G2011, [d-Asp^3^, (*E*)-Dhb^7^]MC-RR for *Planktothrix* G2020, Ferintoic acid B for *Microcystis* G2020, and Oscillamide Y for overall cyanopeptide production by all three species.

**Figure 3:**
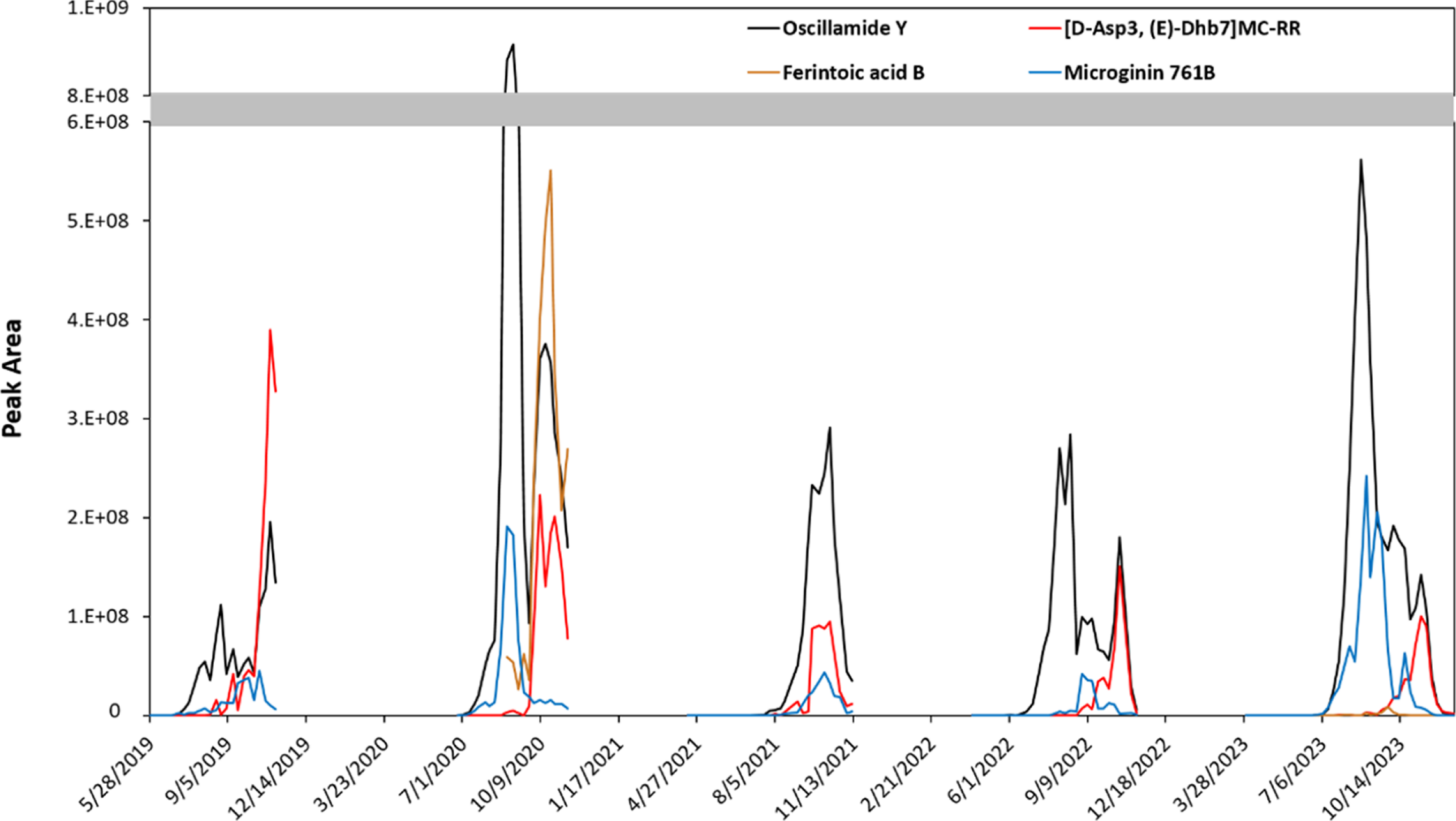
Time series of indicator metabolites: Oscillamide Y (black) produced by all three species from Lake Greifensee, [d-Asp^3^, (*E*)-Dhb^7^]MC-RR (red) produced by *Planktothrix* G2020 isolate, Microginin 761B (blue) produced by *Microcystis* G2011 isolate, and Ferintoic acid B (yellow ocher) produced by *Microcystis* G2020 isolate (see Figure S12 with *y*-axis in log-scale).

Data in Figure 3 show that Microginin 761B and [d-Asp^3^, (*E*)-Dhb^7^]MC-RR were detected in Lake Greifensee in all five years with comparable detection frequency (Figure 2) for each year, while Ferintoic acid B occurred only in 2020 and 2023. Although all four metabolites showed peak concentrations in late summer or autumn, the exact time of their highest abundance varied. For example, the highest abundance based on the peak area of Micorginin 761B occurred in late August 2023; of Ferintoic acid B in late September; and of [d-Asp^3^, (*E*)-Dhb^7^]MC-RR in November. The peak abundance of indicator metabolites suggests a possible shift in dominance or metabolite productivity of the producing strains across the season.

### Implications

Monitoring cyanobacterial blooms and determining the risks they pose to human and ecosystem health remains challenging for the scientific community and local authorities. Those metabolites that occur frequently and in high abundance, i.e., those that are comparatively easy to detect at the onset of bloom development, should be prioritized. We demonstrate that we face complex mixtures of compounds and that bioactive metabolites other than WHO toxins can dominate. Blooms composed of one dominating cyanobacterial strain can be predicted, in part, based on seasonal patterns and high autocorrelation, while blooms composed of multiple cyanobacterial species are more challenging to predict due to limited stochastic drivers.^46^ Herein, we present an approach based on indicator metabolites to elucidate the contribution of multiple species to harmful cyanobacterial blooms. Indicator metabolites can either be specific for individual, albeit co-occurring cyanobacterial species or may be representative for an ensemble of cyanobacteria that coproduce a common metabolite. The concept of indicator metabolites can be a complementary measure to the analysis of WHO toxins and cyanobacterial abundance (cell count) to monitor for harmful cyanobacterial blooms. Together, these ecological and chemical parameters may also reveal new insights into how drivers such as species abundance and co-occurrence relate to metabolite production rates, i.e., *in situ* cell quotas.

## Supporting Information Available

Detailed methodology and supporting data in Text S1, Tables S1–S6, and Figures S1–S12 (PDF)
Documented identification of metabolite structures by MS^2^ spectra annotation (XLSX)
