# Supplementary material for: Tracking Extensive Portfolio of Cyanotoxins in Five-Year Lake Survey and Identifying Indicator Metabolites of Cyanobacterial Taxa
Source: Environ Sci Technol. 2024 Aug 30;58(37):16560–9. doi: 10.1021/acs.est.4c04813 (PMC11411708; doi:10.1021/acs.est.4c04813)
Supplement: Supplementary file 1 — es4c04813_si_001.pdf [file es4c04813_si_001.pdf]

Tracking extensive portfolio of cyanotoxins in five-year lake survey and identifying indicator metabolites of cyanobacterial taxa

Xuejian Wang,[1] Simon Wullschleger,[1] Martin Jones,[1,2] Marta Reyes [1], Raphael Bossart[1], Francesco Pomati,[1] Elisabeth M.-L. Janssen[1*]

[1] Swiss Federal Institute of Aquatic Science and Technology (EAWAG), Dübendorf, 8600, Switzerland.

[2] School of Biosciences, University of Birmingham, Edgbaston, Birmingham, B15 2TT, United Kingdom

*Corresponding author: Email: elisabeth.janssen@eawag.ch

Supporting Information

The electronic supporting information contains 33 pages including 1 text, 6 tables and 12 figures.

List of Text

Text S1.
Materials…………………………………………………………………….……..S3

List of Tables

Table S1. Reference standards and bioreagents…………………………………………….. S4

Table S2. Genetic analysis for 16S rRNA and PCR analysis…………………………......S5

Table S3. Tentative candidates of metabolites with confidence level 3…………………..…S6

Table S4. Detection frequency of metabolite class in biomass………………………………S6

Table S5. Spearman's rank coefficient of cyanobacterial metabolites and monitoring parameters……………………………………………………………………….……S7

Table S6. Spearman's rank coefficient of cyanobacterial metabolites………………………S8

List of figures

Figure S1. Microscope images of two cyanobacteria isolates………………………………S9

Figure S2. Time-series of all cyanobacterial metabolites that could be quantified with available reference materials……………………………………………….…………..S10

Figure S3. Violine plots showing the concentration range of target metabolites (ng/L) in biomass samples and aqueous samples……………………………………………...S14

Figure S4. Time-series of all cyanobacterial metabolites for which no reference materials were available…………………………………………………………………………S15

Figure S5. Violin plots showing the peak area range of cyanobacterial metabolites in the aqueous samples…………………………………………………………………….S28

Figure S6. Detection frequency (%) of cyanobacterial metabolites in aqueous samples…..S28

Figure S7. Co-variance plot of Chl-a, phycocyanin, phycocyanin/Chl-a with [D-Asp^3, (*E*)-Dhb^7]MC-RR and Oscillamide Y……......................................……………………....S29

Figure S8. Time-series of indicator metabolites, Chl-a and phycocyanin…………….…S30

Figure S9. Principal component analysis of indicator metabolites and monitoring parameters…………………………………………............……………………S31

Figure S10. Time-series of phytoplankton abundance……………………………......S32

Figure S11. Analogous to Figure 2 in the main manuscript with changed colors to accommodate color-blindness……………………………………………………….…S33

Figure S12. Time-series of indicator metabolites (identical to Figure 3 but with y-axis in log-scale)……………………………………………………………………………...S33

Text S1

Materials. Microcystin reference standards MC-LR, MC-YR, MC-RR, MC-LF, MC-LA, MC-LW, MC-LY, and Nodularin-R (all >95% purity by HPLC) were obtained from Enzo Life Science (Lausen, Switzerland) and [D-Asp^3, (*E*)-Dhb^7] MC-RR (>95% purity by HPLC) from CyanoBiotech GmbH (Berlin, Germany). Bioreagents for Aeruginosin 98B, Cyanopeptolin A, Cyanopeptolin D, Anabaenopeptin A, Anabaenopeptin B, and Oscillamide Y (all >90% purity by HPLC) were obtained from CyanoBiotech. Aerucyclamide A was obtained as purified bioreagent in dimethyl sulfoxide by Prof. Karl Gademann (University Zurich, Switzerland).

Composition of modified WC growth medium (Guillard et al 1972).

Components	Concentration (mg L^{-1})
$K_2HPO_4.3H_2O$	11.4
$NaNO_3$	85
$CaCl_2.2H_2O$	36.8
$MgSO_4.7H_2O$	37
$NaHCO_3$	12.6
Na_2EDTA	4.36
$FeCl_3.6H_2O$	3.15
$CuSO_4.5H_2O$	0.01
$ZnSO_4.7H_2O$	0.022
$CoCl_2.6H_2O$	0.01
$MnCl_2.4H_2O$	0.18
$Na_2MoO_4.2H_2O$	0.006
H_3BO_3	1.00
TES buffer	115

Guillard, R.R. and C.J. Lorenzen, Yellow-Green Algae with Chlorophyllide C. Journal of Phycology, 1972. 8(1): p. 10-&.

Table S1. Reference standards and bioreagents used in the metabolite analysis with their molecular formula, monoisotopic mass, and dominant precursor ion form with associated mass-to-charge (m/z) ratio.

metabolite	molecular formula	Monoisotopic mass (Da)	dominant precursor ion and *m*/*z* value	
Aerucyclamide A	$C_{24}H_{34}N_6O_4S_2$	534.20830	$[M+H]^+$	535.21557
Anabaenopeptin A	$C_{44}H_{57}N_7O_{10}$	843.41669	$[M+H]^+$	844.42397
Anabaenopeptin B	$C_{41}H_{60}N_{10}O_9$	836.45447	$[M+H]^+$	837.46175
Oscillamide Y	$C_{45}H_{59}N_7O_{10}$	857.43234	$[M+H]^+$	858.43962
Cyanopeptolin A	$C_{46}H_{72}N_{10}O_{12}$	956.53312	$[M+H]^+$	957.54039
Cyanopeptolin D	$C_{48}H_{76}N_8O_{12}$	956.55827	$[M+H]^+$	957.56555
MC-LR	$C_{49}H_{74}N_{10}O_{12}$	994.54877	$[M+H]^+$	995.55604
MC-HilR	$C_{50}H_{76}N_{10}O_{12}$	1008.56442	$[M+H]^+$	1009.57169
MC-LA	$C_{46}H_{67}N_7O_{12}$	909.48477	$[M+H]^+$	910.49205
MC-LF	$C_{52}H_{71}N_7O_{12}$	985.51607	$[M+H]^+$	986.52335
MC-LW	$C_{54}H_{72}N_8O_{12}$	1024.52697	$[M+H]^+$	1025.53425
MC-LY	$C_{52}H_{71}N_7O_{13}$	1001.51099	$[M+H]^+$	1002.51826
MC-RR	$C_{49}H_{75}N_{13}O_{12}$	1037.56581	$[M+2H]^{2+}$	519.79018
MC-YR	$C_{52}H_{72}N_{10}O_{13}$	1044.52803	$[M+H]^+$	1045.53531
[D-Asp^3,(*E*)-Dhb^7]MC-RR	$C_{48}H_{73}N_{13}O_{12}$	1023.55016	$[M+2H]^{2+}$	512.78236
[D-Asp^3]MC-LR	$C_{48}H_{72}N_{10}O_{12}$	980.53312	$[M+H]^+$	981.54039
Nodularin-R	$C_{41}H_{60}N_8O_{10}$	824.44324	$[M+H]^+$	825.45052
Aeruginosin 98B	$C_{29}H_{46}N_6O_9S$	654.30470	$[M+H]^+$	655.31197

Table S2. Genetic analysis for 16S rRNA and presence of microcystin mcyE and anatoxin anaC genes by PCR analysis for three isolated cyanobacteria strains from lake Greifensee.

		PCR result		16S RNA BLAST result (top hits)			
Strain name	Isolated year	*mcyE*	*anaC*	Scientifc name	Max score	Per. Ident	Hit Nbr with similar name and same score
Microcystis G2011	2011	negative	negative	*Microcystis aeruginosa*	1136	100.00%	32
				Microcystis smithii	1136	100.00%	1
				Microcystis flos-aquae	1136	100.00%	1
Planktothrix G2020	2020	positive	negative	*Planktothrix agardhii*	1138	100.00%	7*
				Planktothrix rubescens	1133	99.84%	1*
				Planktothrix rubescens	1131	99.68%	1
Microcystis G2020	2020	negative	negative	*Microcystis aeruginosa*	1133	99.84%	12
				Synechocystis sp.	1133	99.84%	1

* *Planktothrix* G2020 showed high similarity in dominant sequence with *Planktothrix agardhii*, yet the background copy (not shown) suggested *Planktothrix rubescens.*

Table S3. Tentative candidates of metabolites with confidence level 3 where the MS^2 annotation agrees in most parts but did not provide unequivocal evidence to support higher confidence identification.

No.	Protein Name	Molecule Name	Molecule Formula
1	Anabaenopeptin	Lyngbyaureidamide B; Anabaenopeptin NZ841; Nostamide A	C45H59N7O9
2	Cyclamide	Bistratamide I	C25H36N6O6S
3	Microginin	Microginin 612; Spumigin J	C32H44N4O8
4	Microcystin	MC-OiaR	C54H73N11O13
5	Microcystin	MC-MhtyR; MC-AhppaR	C54H76N10O13
6	Microcystin	[Mser7]MC-RR	C49H77N13O13
7	Microcystin	[D-Asp^3]MC-EE; [D-Asp^3,Dha^7]MC-EE(OMe)	C46H63N7O16
8	Microcystin	[D-Leu^1,Mdha-Cys(O)^7]MC-LR	C55H87N11O15S
9	Microcystis	[D-Leu^1,Mdha-Cys^7]MC-LR	C55H87N11O14S
10	Anabaenopeptin	Anabaenopeptin KB906; Anabaenopeptin 906B1	C46H70N10O9
11	Anabaenopeptin	Brunsvicamide B	C46H66N8O8

Table S4. Detection frequency of each metabolite class in biomass samples from the 5-year sample campaign in (2019-2023) in Lake Greifensee, Switzerland.

	2019	2020	2021	2022	2023	sum
# of sampling dates	24	21	28	30	35	138
			detection frequency			sum
Anabaenopeptins	17%	15%	20%	17%	25%	95%
Microcystins	16%	9%	12%	14%	18%	70%
Microginins	17%	15%	20%	17%	25%	94%
Others	12%	7%	10%	10%	9%	49%

Table S5. Spearman's rank correlation coefficients for cyanobacterial metabolites and monitoring parameters in Lake Greifensee across data of the 5-year sampling campaign from 2019-2023. Monitoring parameters: Chl-a and phycocyanin measured by fluorimetry (TriLux, Chelsea Technologies Ltd, Surrey, UK); Oxygen, water temperature and pH, measured using a multi-parameter CTD probe (OCEAN SEVEN 316Plus); total phosphorus, measured by spectrophotometry (DIN EN ISO 6878, 2004); and total nitrogen, measured by spectrophotometry (catalytic combustion at 720 °C and analysis of NOx by chemiluminescence). The monitoring parameters were acquired at the same location and depth (3 m) as samples used for cyanobacterial metabolite analyses.

	Total phosphorus	Total nitrogen	Temperature	Oxygen	pH	Chl-a	Phycocyanin	Phycocyanin/Chl-a
Oscillamide Y	-0.12	-0.29	0.28	-0.12	-0.08	0.21	0.45	0.5
Anabaenopeptin A	-0.11	-0.32	0.22	-0.15	-0.06	0.21	0.44	0.47
Anabaenopeptin B	-0.14	-0.25	0.3	-0.05	-0.07	0.23	0.45	0.48
[D-Asp3_E-Dhb7]MC-RR	0.04	-0.36	-0.35	-0.45	0.03	-0.05	0.31	0.54
MC-LR	0.03	-0.43	0.36	-0.05	-0.01	0.23	0.5	0.55
[D-Asp3]MCLR	0.01	-0.49	-0.21	-0.47	0.07	-0.08	0.29	0.54
MC-LA	0.15	-0.31	0.32	-0.08	-0.02	0.32	0.46	0.34
MC-RR	0.2	-0.5	0.3	-0.18	-0.11	0.19	0.27	0.33
MC-YR	-0.06	-0.47	0.18	-0.1	0.1	0.02	0.39	0.61
Anabaenopeptin F	-0.13	-0.27	0.33	-0.02	-0.06	0.24	0.47	0.49
Anabaenopeptin D	-0.07	-0.3	-0.04	-0.27	0.23	0.28	0.44	0.26
Anabaenopeptin 871	0.05	-0.22	-0.07	-0.2	0.08	0.32	0.54	0.41
Anabaenopeptin J	-0.15	-0.25	-0.07	-0.26	0.15	0.19	0.36	0.26
Nodulapeptin 865	-0.05	-0.54	-0.21	-0.46	0.21	-0.13	0.26	0.61
Anabaenopeptin 807	-0.1	-0.05	-0.02	-0.13	0.06	0.25	0.43	0.25
Anabaenopeptin 820	-0.12	-0.39	0.28	-0.21	0.1	0.19	0.23	0.17
Anabaenopeptin C	0.14	-0.05	-0.1	-0.17	-0.16	0.12	0.12	0.15
Anabaenopeptin NZ825	-0.13	0.02	-0.03	-0.08	0.13	0.21	0.43	0.26
Ferintoic acid A	-0.13	-0.04	-0.13	-0.12	0.2	0.06	0.39	0.32
Ferintoic acid B	-0.12	0.01	-0.04	-0.09	0.12	0.2	0.43	0.27
Nodulapeptin 821	-0.13	0.02	-0.04	-0.07	0.13	0.21	0.43	0.26
Nodulapeptin 855b	-0.08	0	0.02	-0.08	0.06	0.26	0.44	0.23
MC-KynA	0.02	-0.03	-0.04	-0.13	-0.21	0.22	0.33	0.28
[NMe-Ala7]MC-LR	0.1	-0.48	0.13	-0.33	0.06	0.12	0.19	0.17
MC-(H2)YA	0.02	-0.4	-0.06	-0.24	0.18	-0.01	0.14	0.29
[D-Leu1]MC-HphR	0.09	-0.32	0	-0.13	0.11	0.13	0.3	0.28
Microginin 580	0.05	-0.19	-0.14	-0.24	0.23	0.26	0.41	0.21
Microginin 757	-0.11	0.02	-0.1	0.03	-0.03	0.1	0.47	0.62
Microginin 791	-0.11	0.05	-0.16	0.01	-0.02	0.08	0.44	0.58
Oscillaginin A	0.01	-0.21	-0.11	-0.21	0.17	0.27	0.45	0.24
Microginin FR5	0.23	-0.49	-0.31	-0.49	0.05	0.06	0.29	0.45
Microginin 761B	-0.03	-0.23	0.23	-0.12	-0.15	0.29	0.45	0.39
Planktocyclin	-0.02	-0.41	-0.42	-0.51	0.16	-0.19	0.21	0.51
Planktopeptin BL1125	-0.03	-0.35	-0.39	-0.43	0.05	-0.17	0.22	0.53
Aeruginosamide	0.02	-0.2	-0.06	-0.03	-0.03	-0.1	0.23	0.53

Table S6. Spearman's rank correlation coefficients for cyanobacterial metabolites detected across the 5-year sampling campaign (2019-2023) at lake Greifensee, Switzerland.

	Oscillamide Y	Anabaenopeptin A	Anabaenopeptin B	[D-Asp3_E-Dhb7]MC-RR	MC-LR	[D-Asp3]MCLR	MC-LA	MC-RR	MC-YR	Anabaenopeptin F	Anabaenopeptin D	Anabaenopeptin 871	Anabaenopeptin J	Nodulapeptin 865	Anabaenopeptin 807	Anabaenopeptin 820	Anabaenopeptin C	Anabaenopeptin NZ825	Ferintoic acid A	Ferintoic acid B	Nodulapeptin 821	Nodulapeptin 855b	MC-KynA	[NMe-Ala7]MC-LR	MC-(H2)YA	[D-Leu1]MC-HphR	Microginin 580	Microginin 757	Microginin 791	Oscillaginin A	Microginin FR5	Microginin 761B	Planktocyclin	Planktopeptin BL1125	Aeruginosamide
Oscillamide Y	1.00	0.98	0.96	0.63	0.64	0.66	0.43	0.46	0.58	0.95	0.72	0.74	0.66	0.42	0.63	0.63	0.30	0.54	0.42	0.55	0.54	0.58	0.71	0.24	0.09	0.06	0.53	0.66	0.63	0.61	0.53	0.85	0.51	0.54	0.36
Anabaenopeptin A	0.98	1.00	0.97	0.67	0.64	0.68	0.42	0.47	0.58	0.95	0.74	0.74	0.68	0.45	0.65	0.67	0.32	0.55	0.43	0.55	0.55	0.58	0.74	0.19	0.03	0.01	0.55	0.65	0.63	0.62	0.54	0.86	0.55	0.58	0.38
Anabaenopeptin B	0.96	0.97	1.00	0.59	0.68	0.62	0.46	0.50	0.60	0.97	0.67	0.67	0.60	0.43	0.56	0.67	0.33	0.49	0.38	0.49	0.49	0.52	0.67	0.18	0.04	0.01	0.46	0.67	0.64	0.54	0.49	0.83	0.47	0.51	0.41
[D-Asp3_E-Dhb7]MC-R	0.63	0.67	0.59	1.00	0.49	0.90	0.28	0.44	0.53	0.57	0.55	0.66	0.57	0.70	0.47	0.32	0.30	0.37	0.36	0.38	0.36	0.33	0.61	0.23	0.26	0.17	0.54	0.76	0.77	0.59	0.80	0.62	0.91	0.93	0.60
MC-LR	0.64	0.64	0.68	0.49	1.00	0.60	0.73	0.81	0.84	0.68	0.41	0.47	0.39	0.50	0.26	0.54	0.19	0.28	0.21	0.28	0.28	0.28	0.31	0.45	0.32	0.29	0.28	0.49	0.45	0.33	0.50	0.57	0.36	0.40	0.54
[D-Asp3]MCLR	0.66	0.68	0.62	0.90	0.60	1.00	0.32	0.55	0.66	0.59	0.54	0.62	0.53	0.76	0.40	0.35	0.28	0.27	0.33	0.28	0.27	0.26	0.52	0.25	0.28	0.20	0.45	0.69	0.69	0.49	0.81	0.57	0.86	0.88	0.63
MC-LA	0.43	0.42	0.46	0.28	0.73	0.32	1.00	0.64	0.46	0.51	0.37	0.37	0.30	0.31	0.26	0.55	-0.01	0.25	0.12	0.24	0.25	0.28	0.13	0.52	0.34	0.30	0.35	0.27	0.24	0.38	0.27	0.51	0.17	0.14	0.22
MC-RR	0.46	0.47	0.50	0.44	0.81	0.55	0.64	1.00	0.66	0.48	0.26	0.37	0.21	0.46	0.05	0.42	0.33	-0.02	0.00	-0.02	-0.02	0.01	0.27	0.60	0.38	0.30	0.16	0.32	0.29	0.22	0.60	0.54	0.30	0.36	0.55
MC-YR	0.58	0.58	0.60	0.53	0.84	0.66	0.46	0.66	1.00	0.57	0.33	0.40	0.31	0.59	0.16	0.41	0.13	0.18	0.27	0.17	0.18	0.11	0.22	0.35	0.35	0.30	0.19	0.49	0.46	0.23	0.51	0.39	0.46	0.49	0.60
Anabaenopeptin F	0.95	0.95	0.97	0.57	0.68	0.59	0.51	0.48	0.57	1.00	0.68	0.65	0.58	0.42	0.55	0.70	0.22	0.46	0.34	0.45	0.46	0.49	0.61	0.17	0.05	0.03	0.47	0.65	0.62	0.56	0.42	0.83	0.46	0.49	0.40
Anabaenopeptin D	0.72	0.74	0.67	0.55	0.41	0.54	0.37	0.26	0.33	0.68	1.00	0.86	0.86	0.40	0.76	0.56	0.14	0.66	0.51	0.67	0.66	0.70	0.64	0.33	0.10	0.03	0.88	0.47	0.47	0.88	0.51	0.70	0.52	0.47	0.18
Anabaenopeptin 871	0.74	0.74	0.67	0.66	0.47	0.62	0.37	0.37	0.40	0.65	0.86	1.00	0.80	0.40	0.74	0.40	0.31	0.65	0.50	0.66	0.65	0.69	0.74	0.35	0.15	0.08	0.85	0.65	0.64	0.88	0.67	0.74	0.57	0.56	0.36
Anabaenopeptin J	0.66	0.68	0.60	0.57	0.39	0.53	0.30	0.21	0.31	0.58	0.86	0.80	1.00	0.31	0.86	0.54	0.14	0.75	0.57	0.76	0.75	0.79	0.67	0.31	0.10	0.00	0.84	0.42	0.42	0.82	0.48	0.61	0.50	0.47	0.17
Nodulapeptin 865	0.42	0.45	0.43	0.70	0.50	0.76	0.31	0.46	0.59	0.42	0.40	0.40	0.31	1.00	0.15	0.34	0.08	0.02	0.10	0.04	0.02	0.00	0.19	0.22	0.41	0.33	0.30	0.55	0.55	0.36	0.60	0.42	0.81	0.76	0.62
Anabaenopeptin 807	0.63	0.65	0.56	0.47	0.26	0.40	0.26	0.05	0.16	0.55	0.76	0.74	0.86	0.15	1.00	0.49	0.18	0.86	0.66	0.87	0.86	0.91	0.74	0.19	-0.13	-0.10	0.79	0.41	0.41	0.77	0.36	0.61	0.38	0.35	0.02
Anabaenopeptin 820	0.63	0.67	0.67	0.32	0.54	0.35	0.55	0.42	0.41	0.70	0.56	0.40	0.54	0.34	0.49	1.00	0.05	0.37	0.21	0.37	0.37	0.41	0.33	0.24	0.02	-0.06	0.43	0.21	0.18	0.44	0.23	0.56	0.26	0.21	0.13
Anabaenopeptin C	0.30	0.32	0.33	0.30	0.19	0.28	-0.01	0.33	0.13	0.22	0.14	0.31	0.14	0.08	0.18	0.05	1.00	0.26	0.29	0.24	0.25	0.22	0.52	0.14	-0.09	-0.06	0.09	0.27	0.27	0.13	0.47	0.35	0.18	0.26	0.18
Anabaenopeptin NZ82	0.54	0.55	0.49	0.37	0.28	0.27	0.25	-0.02	0.18	0.46	0.66	0.65	0.75	0.02	0.86	0.37	0.26	1.00	0.77	0.98	1.00	0.94	0.63	0.23	-0.10	-0.08	0.69	0.39	0.38	0.67	0.27	0.51	0.26	0.23	-0.06
Ferintoic acid A	0.42	0.43	0.38	0.36	0.21	0.33	0.12	0.00	0.27	0.34	0.51	0.50	0.57	0.10	0.66	0.21	0.29	0.77	1.00	0.74	0.76	0.70	0.43	0.20	-0.07	-0.06	0.52	0.32	0.32	0.51	0.32	0.32	0.33	0.30	0.00
Ferintoic acid B	0.55	0.55	0.49	0.38	0.28	0.28	0.24	-0.02	0.17	0.45	0.67	0.66	0.76	0.04	0.87	0.37	0.24	0.98	0.74	1.00	0.98	0.95	0.64	0.21	-0.11	-0.08	0.70	0.39	0.39	0.68	0.28	0.52	0.27	0.24	-0.07
Nodulapeptin 821	0.54	0.55	0.49	0.36	0.28	0.27	0.25	-0.02	0.18	0.46	0.66	0.65	0.75	0.02	0.86	0.37	0.25	1.00	0.76	0.98	1.00	0.94	0.63	0.23	-0.10	-0.08	0.69	0.39	0.38	0.67	0.26	0.51	0.26	0.22	-0.06
Nodulapeptin 855b	0.58	0.58	0.52	0.33	0.28	0.26	0.28	0.01	0.11	0.49	0.70	0.69	0.79	0.00	0.91	0.41	0.22	0.94	0.70	0.95	0.94	1.00	0.68	0.24	-0.11	-0.08	0.72	0.32	0.32	0.71	0.26	0.57	0.22	0.19	-0.11
MC-KynA	0.71	0.74	0.67	0.61	0.31	0.52	0.13	0.27	0.22	0.61	0.64	0.74	0.67	0.19	0.74	0.33	0.52	0.63	0.43	0.64	0.63	0.68	1.00	0.14	-0.17	-0.13	0.60	0.55	0.55	0.62	0.56	0.77	0.43	0.50	0.22
[NMe-Ala7]MC-LR	0.24	0.19	0.18	0.23	0.45	0.25	0.52	0.60	0.35	0.17	0.33	0.35	0.31	0.22	0.19	0.24	0.14	0.23	0.20	0.21	0.23	0.24	0.14	1.00	0.57	0.44	0.34	0.08	0.07	0.38	0.41	0.38	0.13	0.11	0.13
MC-(H2)YA	0.09	0.03	0.04	0.26	0.32	0.28	0.34	0.38	0.35	0.05	0.10	0.15	0.10	0.41	-0.13	0.02	-0.09	-0.10	-0.07	-0.11	-0.10	-0.11	-0.17	0.57	1.00	0.76	0.13	0.14	0.14	0.16	0.29	0.08	0.30	0.28	0.32
[D-Leu1]MC-HphR	0.06	0.01	0.01	0.17	0.29	0.20	0.30	0.30	0.30	0.03	0.03	0.08	0.00	0.33	-0.10	-0.06	-0.06	-0.08	-0.06	-0.08	-0.08	-0.08	-0.13	0.44	0.76	1.00	0.05	0.13	0.13	0.09	0.18	0.10	0.20	0.18	0.18
Microginin 580	0.53	0.55	0.46	0.54	0.28	0.45	0.35	0.16	0.19	0.47	0.88	0.85	0.84	0.30	0.79	0.43	0.09	0.69	0.52	0.70	0.69	0.72	0.60	0.34	0.13	0.05	1.00	0.43	0.43	0.93	0.49	0.58	0.52	0.46	0.15
Microginin 757	0.66	0.65	0.67	0.76	0.49	0.69	0.27	0.32	0.49	0.65	0.47	0.65	0.42	0.55	0.41	0.21	0.27	0.39	0.32	0.39	0.39	0.32	0.55	0.08	0.14	0.13	0.43	1.00	0.99	0.53	0.56	0.65	0.68	0.72	0.63
Microginin 791	0.63	0.63	0.64	0.77	0.45	0.69	0.24	0.29	0.46	0.62	0.47	0.64	0.42	0.55	0.41	0.18	0.27	0.38	0.32	0.39	0.38	0.32	0.55	0.07	0.14	0.13	0.43	0.99	1.00	0.53	0.57	0.64	0.70	0.74	0.62
Oscillaginin A	0.61	0.62	0.54	0.59	0.33	0.49	0.38	0.22	0.23	0.56	0.88	0.88	0.82	0.36	0.77	0.44	0.13	0.67	0.51	0.68	0.67	0.71	0.62	0.38	0.16	0.09	0.93	0.53	0.53	1.00	0.52	0.69	0.54	0.50	0.20
Microginin FR5	0.53	0.54	0.49	0.80	0.50	0.81	0.27	0.60	0.51	0.42	0.51	0.67	0.48	0.60	0.36	0.23	0.47	0.27	0.32	0.28	0.26	0.26	0.56	0.41	0.29	0.18	0.49	0.56	0.57	0.52	1.00	0.53	0.73	0.75	0.57
Microginin 761B	0.85	0.86	0.83	0.62	0.57	0.57	0.51	0.54	0.39	0.83	0.70	0.74	0.61	0.42	0.61	0.56	0.35	0.51	0.32	0.52	0.51	0.57	0.77	0.38	0.08	0.10	0.58	0.65	0.64	0.69	0.53	1.00	0.47	0.50	0.32
Planktocyclin	0.51	0.55	0.47	0.91	0.36	0.86	0.17	0.30	0.46	0.46	0.52	0.57	0.50	0.81	0.38	0.26	0.18	0.26	0.33	0.27	0.26	0.22	0.43	0.13	0.30	0.20	0.52	0.68	0.70	0.54	0.73	0.47	1.00	0.96	0.60
Planktopeptin BL1125	0.54	0.58	0.51	0.93	0.40	0.88	0.14	0.36	0.49	0.49	0.47	0.56	0.47	0.76	0.35	0.21	0.26	0.23	0.30	0.24	0.22	0.19	0.50	0.11	0.28	0.18	0.46	0.72	0.74	0.50	0.75	0.50	0.96	1.00	0.66
Aeruginosamide	0.36	0.38	0.41	0.60	0.54	0.63	0.22	0.55	0.60	0.40	0.18	0.36	0.17	0.62	0.02	0.13	0.18	-0.06	0.00	-0.07	-0.06	-0.11	0.22	0.13	0.32	0.18	0.15	0.63	0.62	0.20	0.57	0.32	0.60	0.66	1.00

(a)

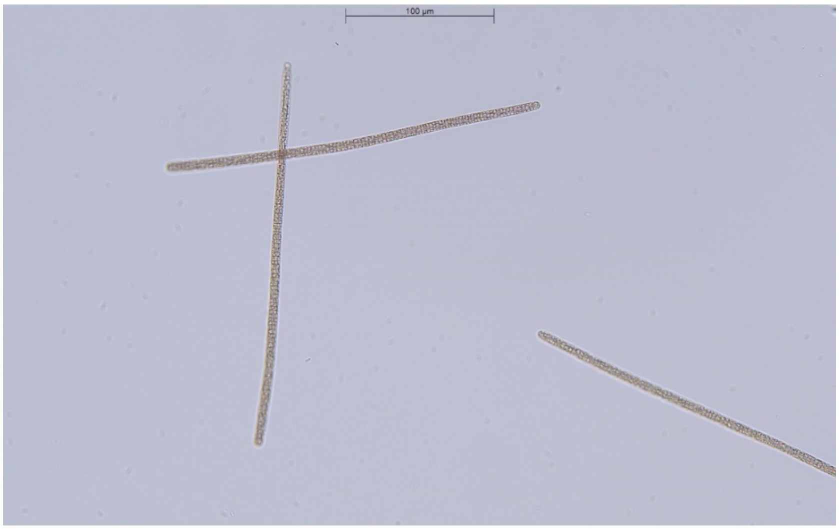

(b)

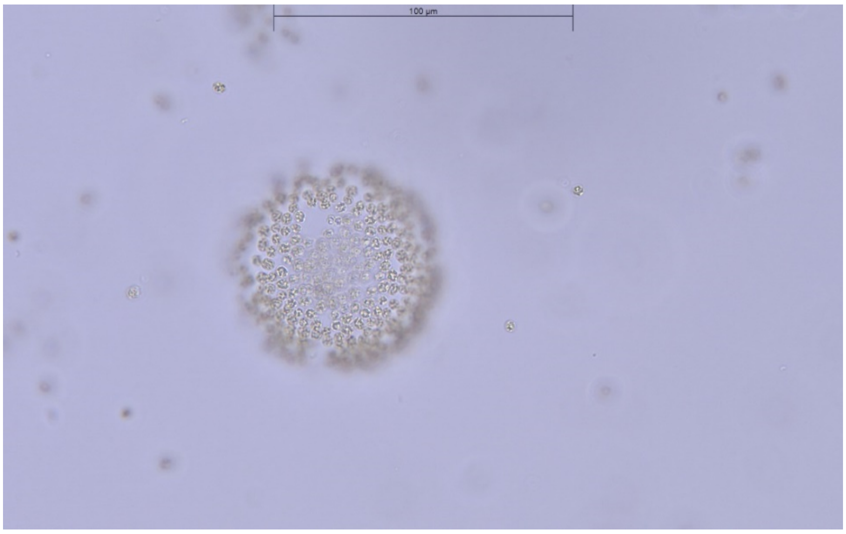

Figure S1. Microscope images of two cyanobacteria isolates from lake Greifensee: (a) *Planktothrix* G2020 and (b) *Microcystis* G2020.

Figure S2. Time-series of seven cyanobacterial metabolites that could be quantified with available reference materials (targets) in water samples (blue) and biomass samples (red) across the 5-year sampling campaign (2019-2023) in Lake Greifensee. Each data point represents one sample out of triplicate samples collected on each sampling date. Solid lines connect the average concentration of these triplicate samples on each sampling date, while white spaces between data sets reflect the dates where no sampling took place. Dashed lines indicate the limit of quantification (LOQ, from extraction through to detection) for each year for water samples and biomass samples.

1. Anabaenopeptin B

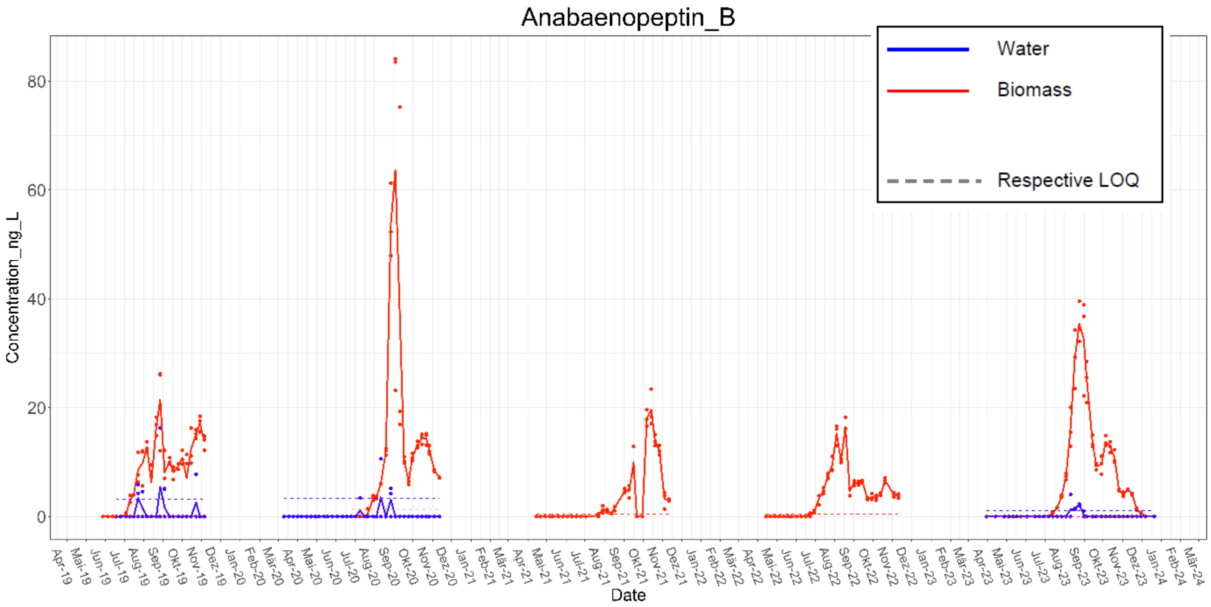

2. Oscillamide Y

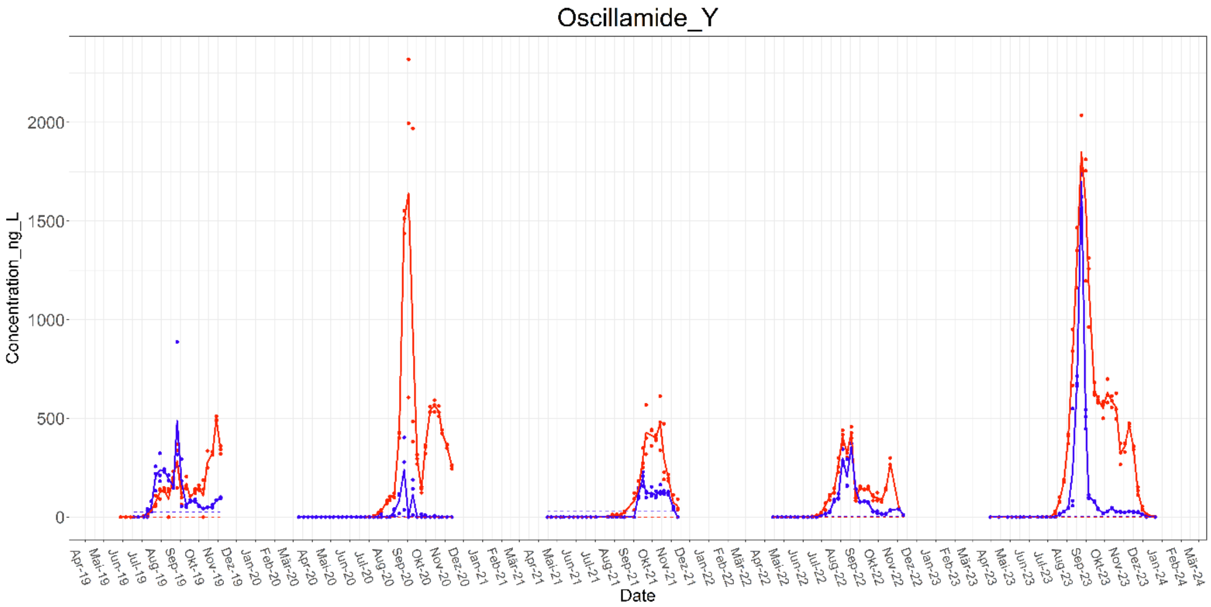

3. [D-Asp^{3}] MC-LR

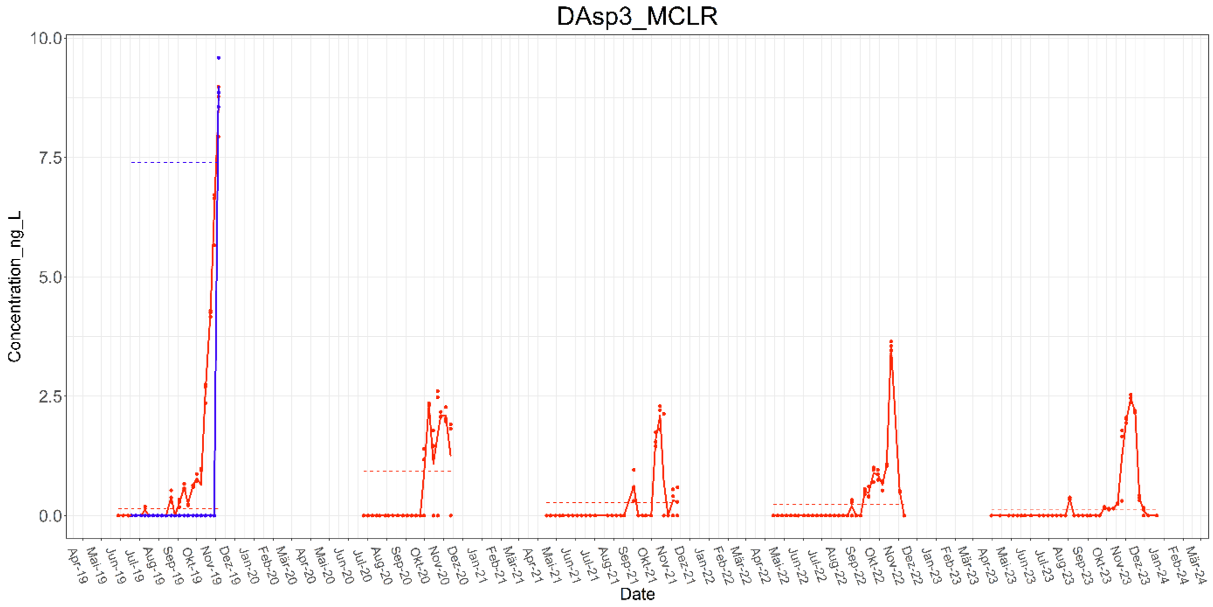

4. MC-LR

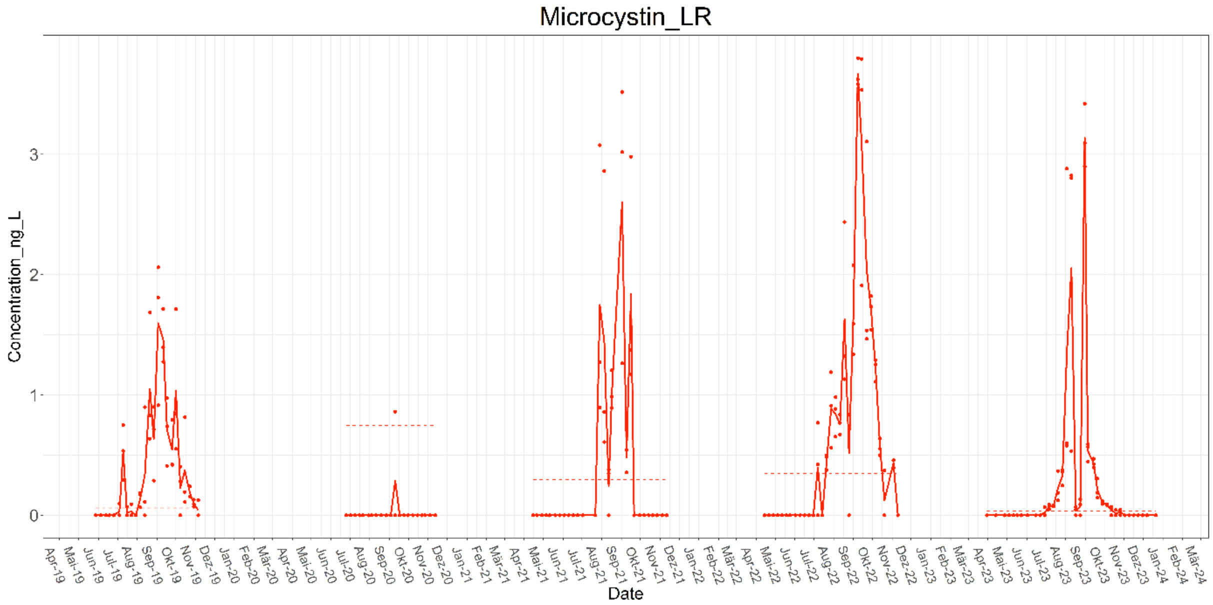

5. MC-LA

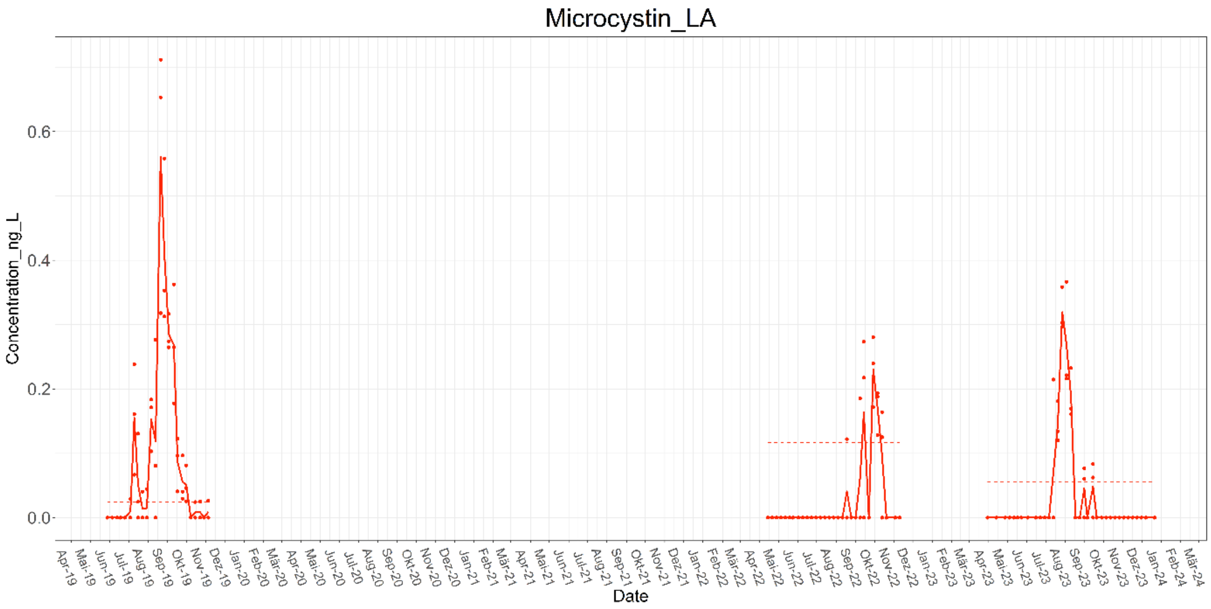
Microcystin_LA
Concentration_ng_L
0.0
0.2
0.4
0.6
Date
Apr-19 Mai-19 Jun-19 Jul-19 Aug-19 Sep-19 Okt-19 Nov-19 Dez-19 Jan-20 Feb-20 Mär-20 Apr-20 Mai-20 Jun-20 Jul-20 Aug-20 Sep-20 Okt-20 Nov-20 Dez-20 Jan-21 Feb-21 Mär-21 Apr-21 Mai-21 Jun-21 Jul-21 Aug-21 Sep-21 Okt-21 Nov-21 Dez-21 Jan-22 Feb-22 Mär-22 Apr-22 Mai-22 Jun-22 Jul-22 Aug-22 Sep-22 Okt-22 Nov-22 Dez-22 Jan-23 Feb-23 Mär-23 Apr-23 Mai-23 Jun-23 Jul-23 Aug-23 Sep-23 Okt-23 Nov-23 Dez-23 Jan-24 Feb-24 Mär-24

6. MC-RR

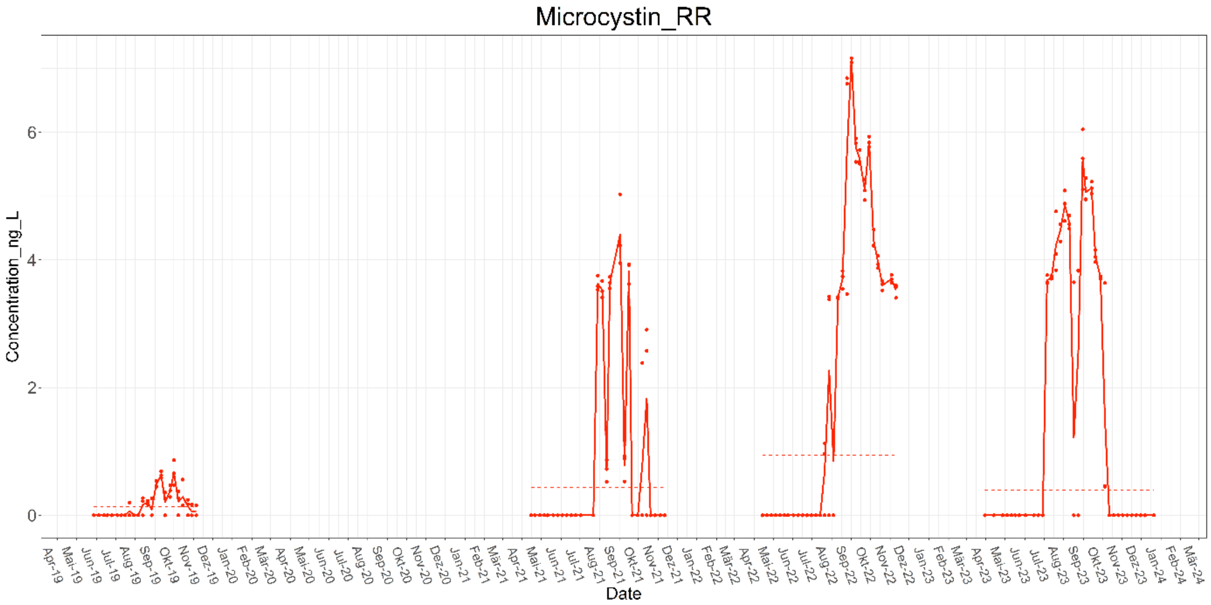
Microcystin_RR
Concentration_ng_L
0
2
4
6
Date
Apr-19 Mai-19 Jun-19 Jul-19 Aug-19 Sep-19 Okt-19 Nov-19 Dez-19 Jan-20 Feb-20 Mär-20 Apr-20 Mai-20 Jun-20 Jul-20 Aug-20 Sep-20 Okt-20 Nov-20 Dez-20 Jan-21 Feb-21 Mär-21 Apr-21 Mai-21 Jun-21 Jul-21 Aug-21 Sep-21 Okt-21 Nov-21 Dez-21 Jan-22 Feb-22 Mär-22 Apr-22 Mai-22 Jun-22 Jul-22 Aug-22 Sep-22 Okt-22 Nov-22 Dez-22 Jan-23 Feb-23 Mär-23 Apr-23 Mai-23 Jun-23 Jul-23 Aug-23 Sep-23 Okt-23 Nov-23 Dez-23 Jan-24 Feb-24 Mär-24

7. MC-YR

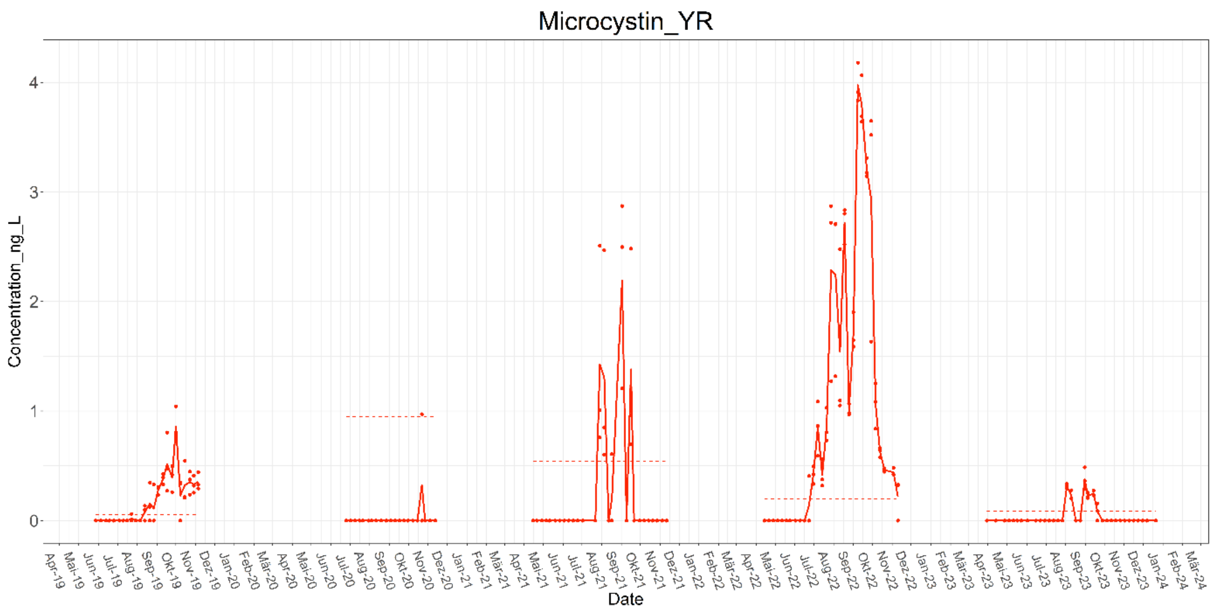
Microcystin_YR
Concentration_ng_L
Date
0
1
2
3
4
Apr-19
Mai-19
Jun-19
Jul-19
Aug-19
Sep-19
Okt-19
Nov-19
Dez-19
Jan-20
Feb-20
Mär-20
Apr-20
Mai-20
Jun-20
Jul-20
Aug-20
Sep-20
Okt-20
Nov-20
Dez-20
Jan-21
Feb-21
Mär-21
Apr-21
Mai-21
Jun-21
Jul-21
Aug-21
Sep-21
Okt-21
Nov-21
Dez-21
Jan-22
Feb-22
Mär-22
Apr-22
Mai-22
Jun-22
Jul-22
Aug-22
Sep-22
Okt-22
Nov-22
Dez-22
Jan-23
Feb-23
Mär-23
Apr-23
Mai-23
Jun-23
Jul-23
Aug-23
Sep-23
Okt-23
Nov-23
Dez-23
Jan-24
Feb-24
Mär-24

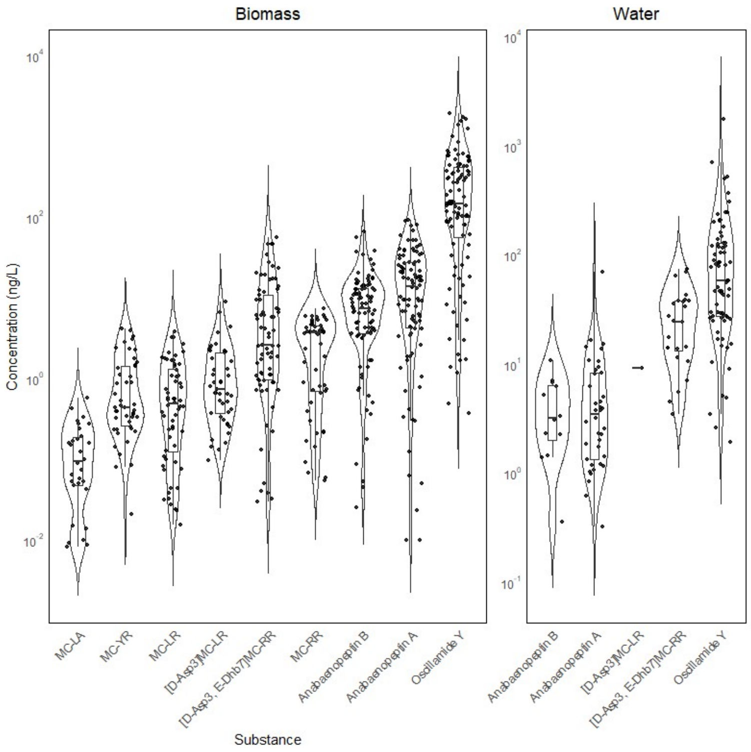

Figure S3. Violin plots showing the concentration range of target metabolites (ng/L) in biomass samples and aqueous samples across the 5-year sampling campaign (2019-2023) in Lake Greifensee for all targeted cyanobacterial metabolites where quantification was possible due to the availability of reference materials.

Figure S4. Time-series of 26 suspected cyanobacterial metabolites for which no reference materials were available (suspects, confidence Level 2) in water samples (blue) and biomass samples (red) across the 5-year sampling campaign (2019-2023) in Lake Greifensee. Solid lines connect the average concentration of these triplicate samples on each sampling date, while white spaces between data sets reflect the dates where no sampling took place.

1. Anabaenopeptin F

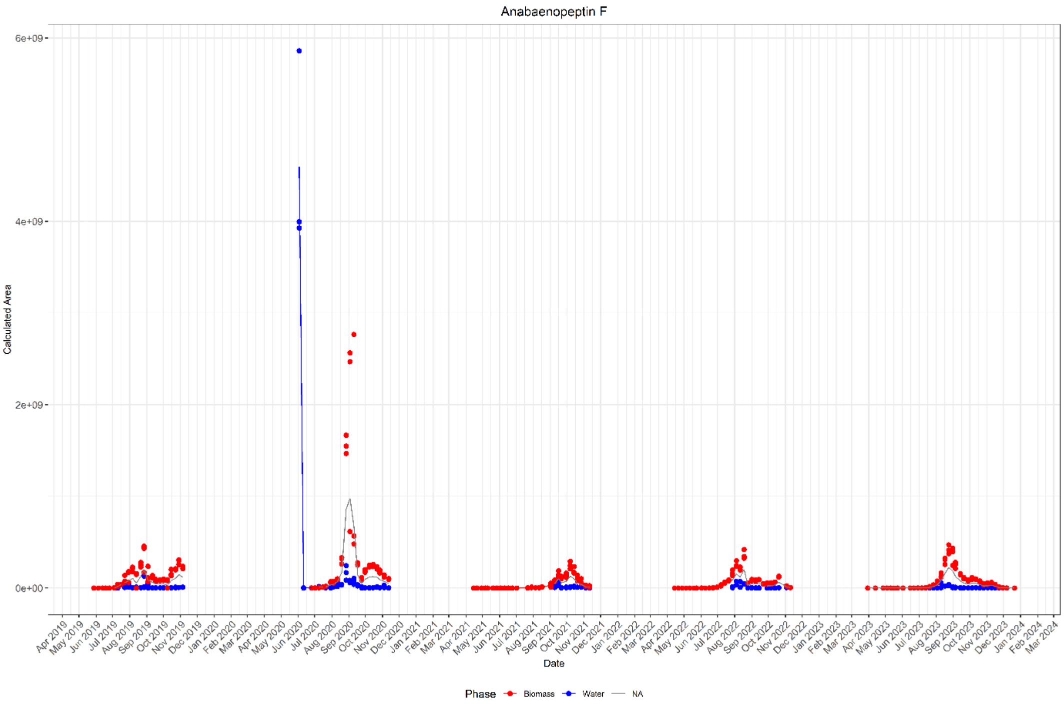

2. Anabaenopeptin D

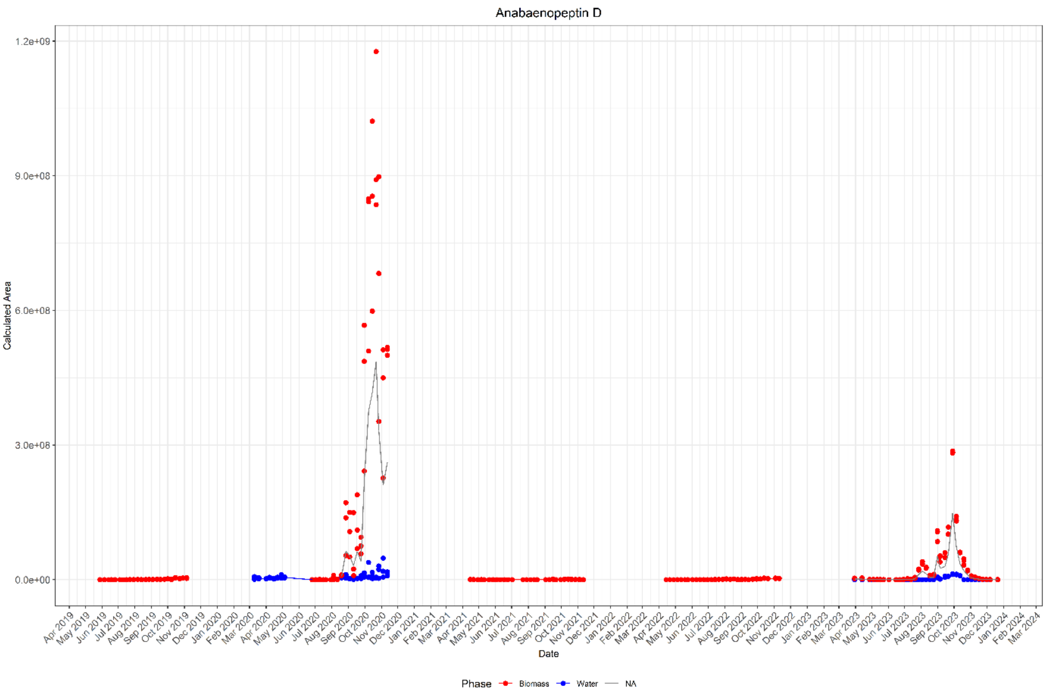

3. Anabaenopeptin 871

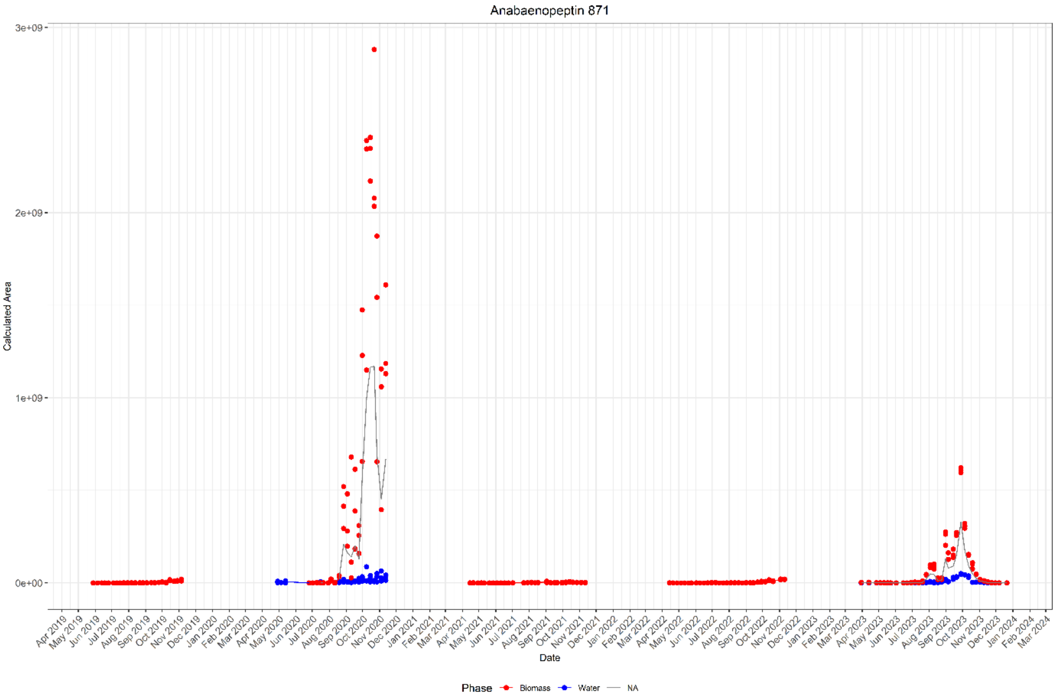

4. Anabaenopeptin J

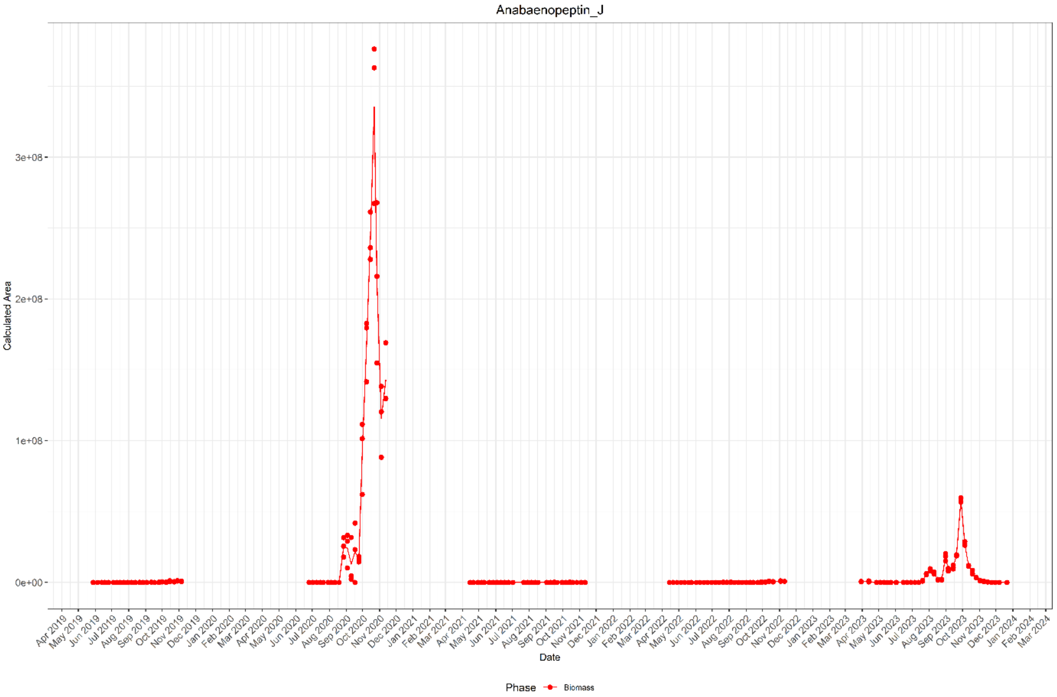

5. Anabaenopeptin 807

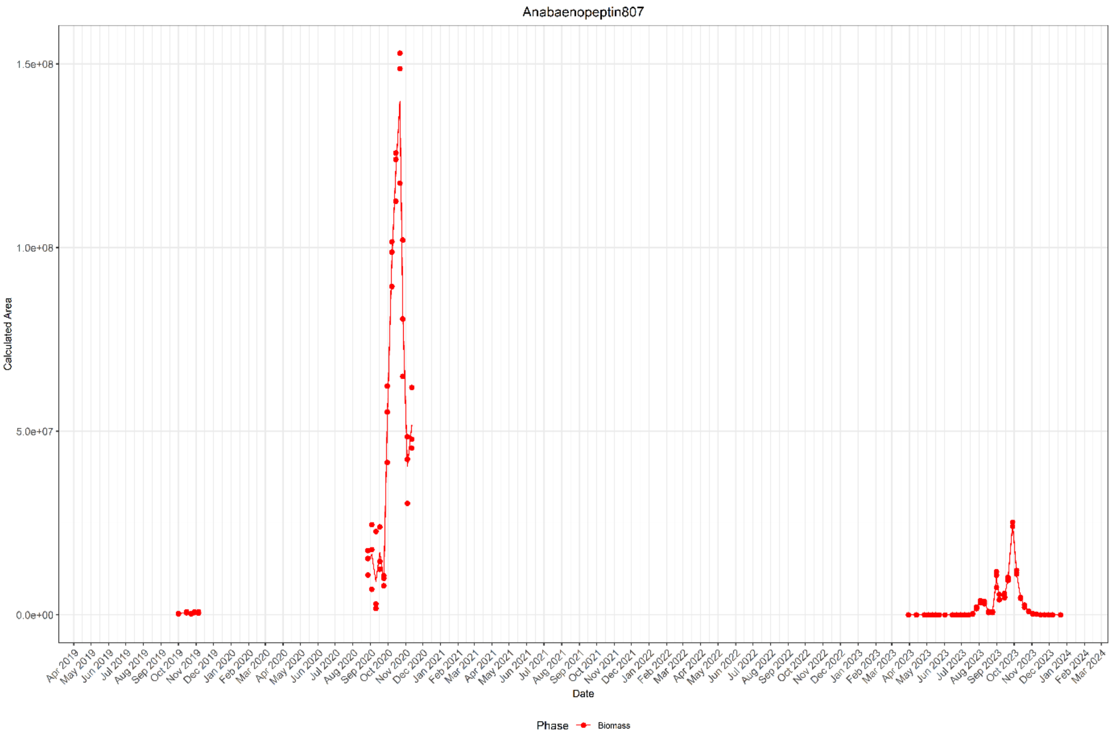

6. Anabaenopeptin 820

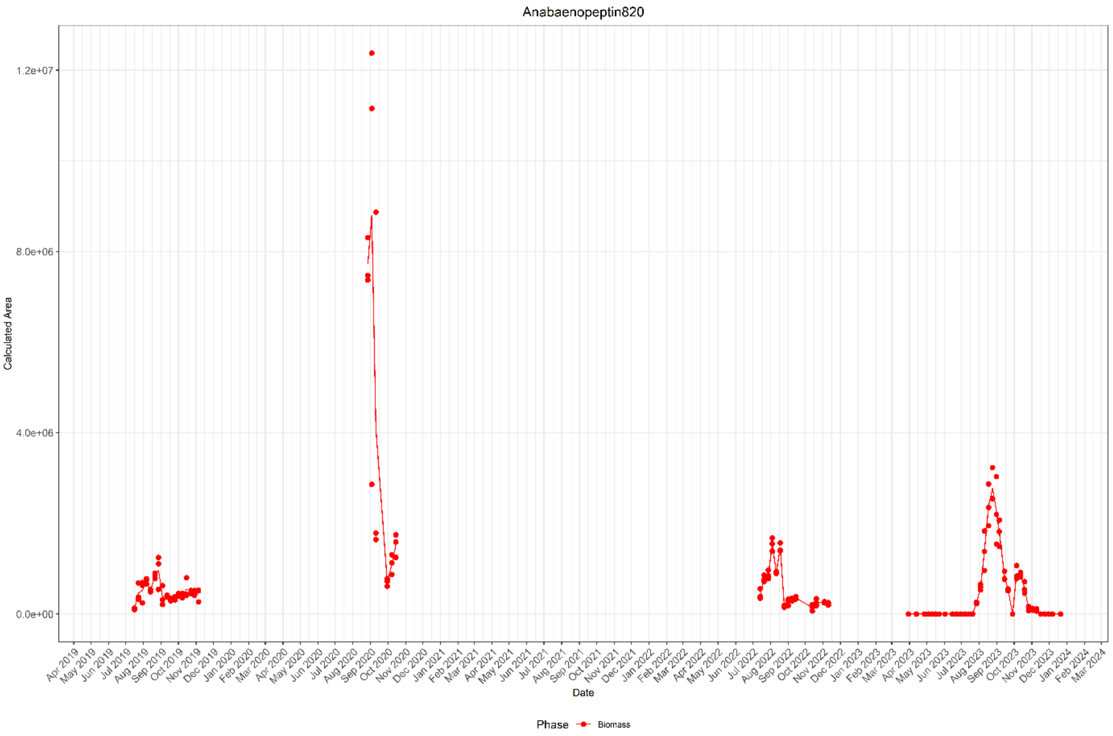

7. Anabaenopeptin C

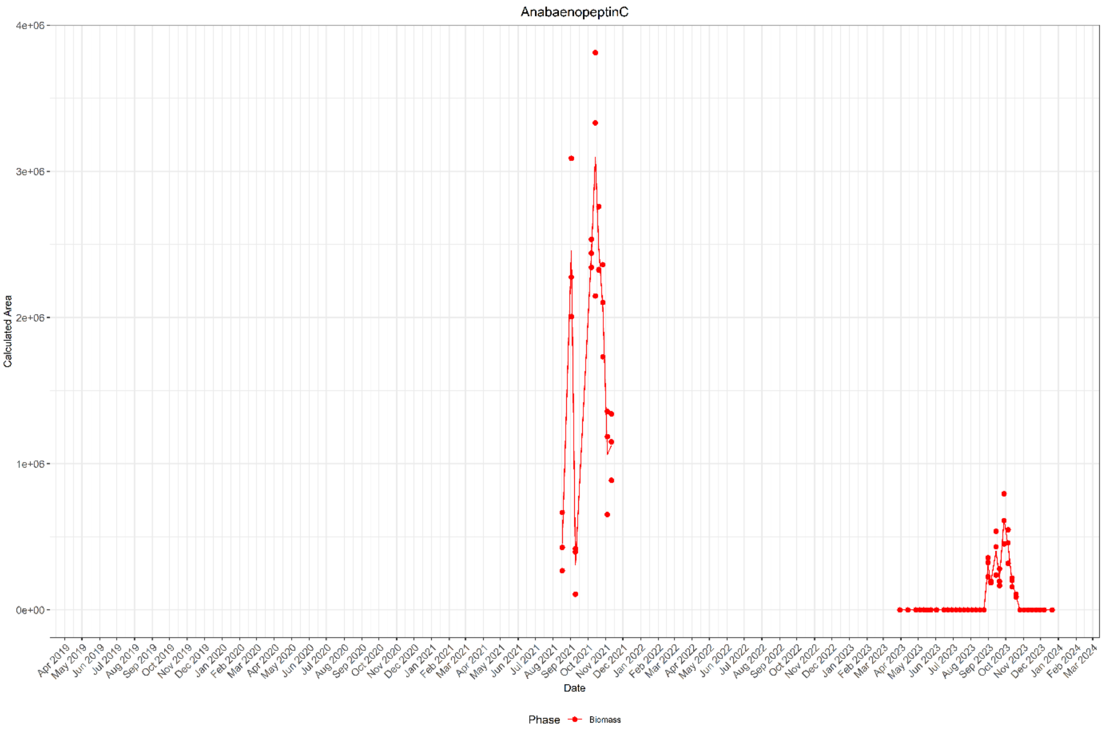

8. Anabaenopeptin NZ825

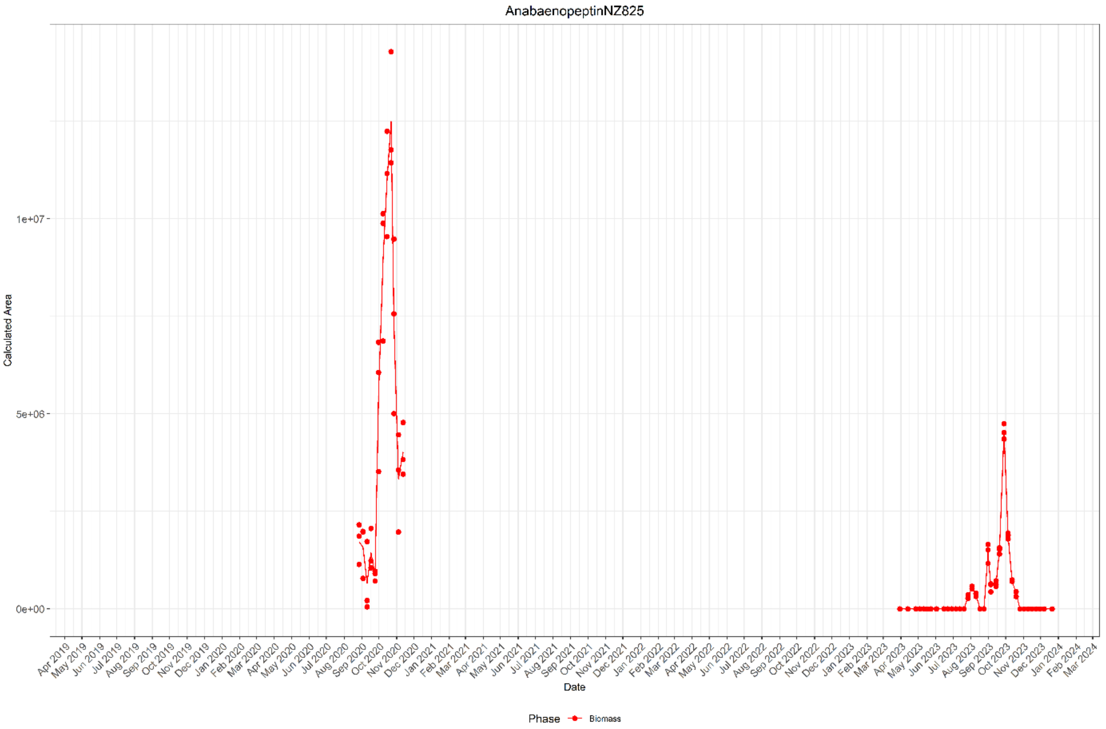

9. Nodulapeptin 865

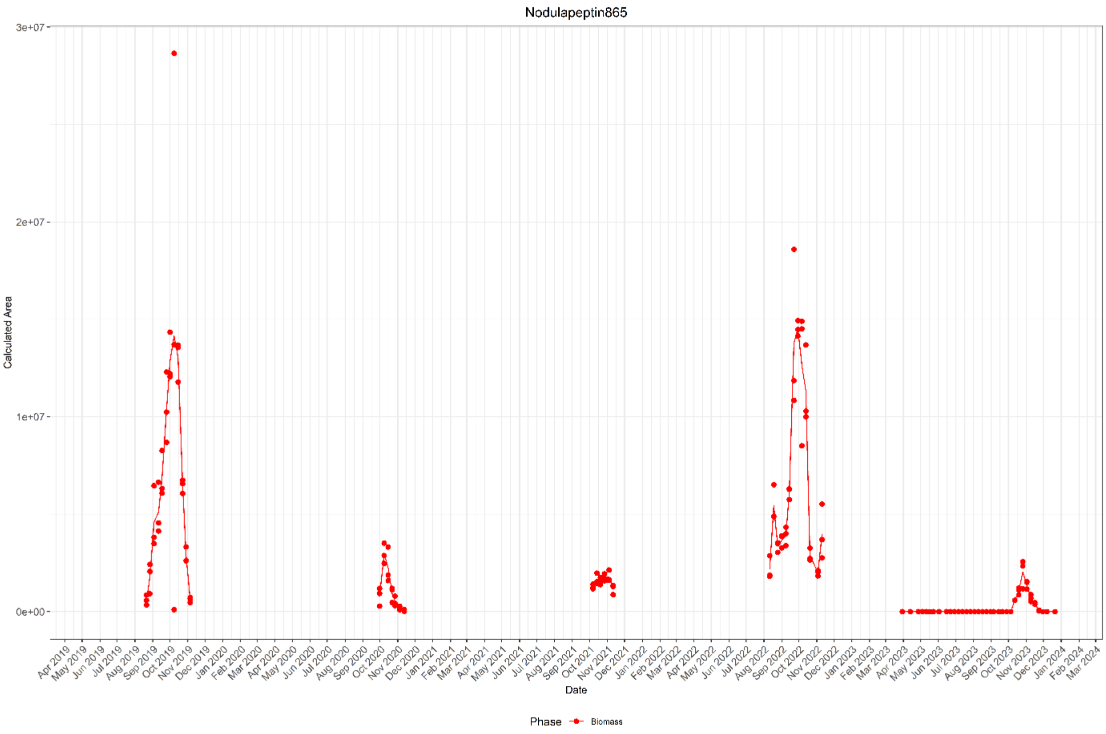

10. Nodulapeptin 821

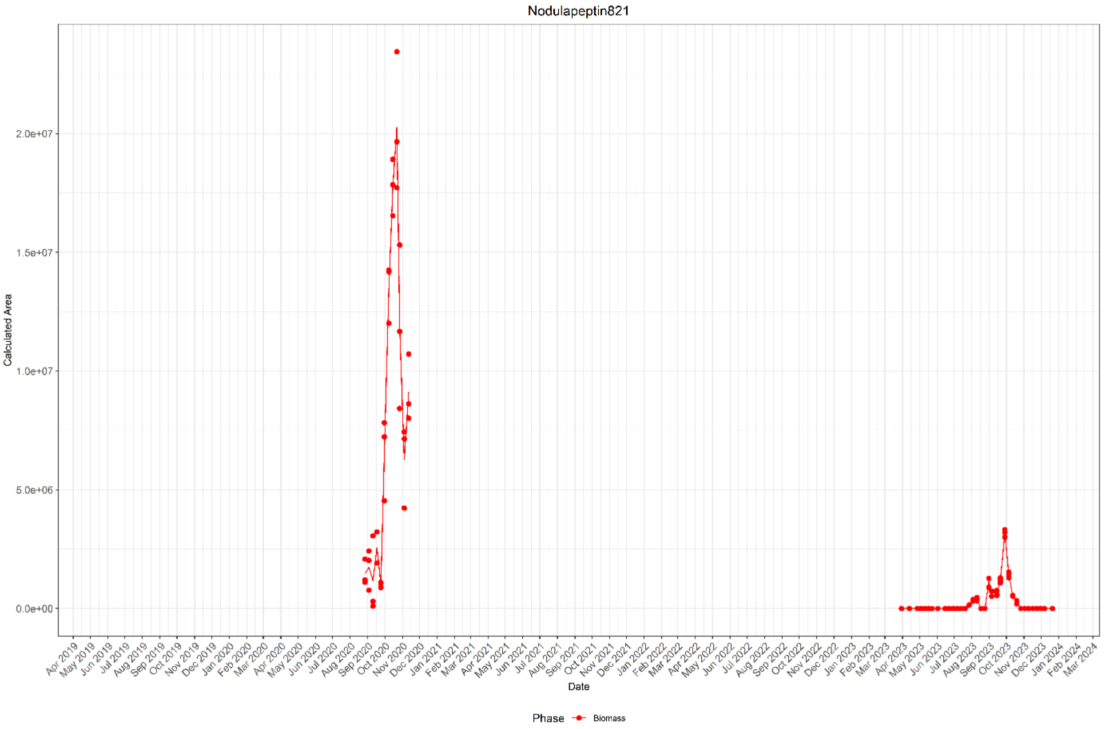

11. Nodulapeptin 855b

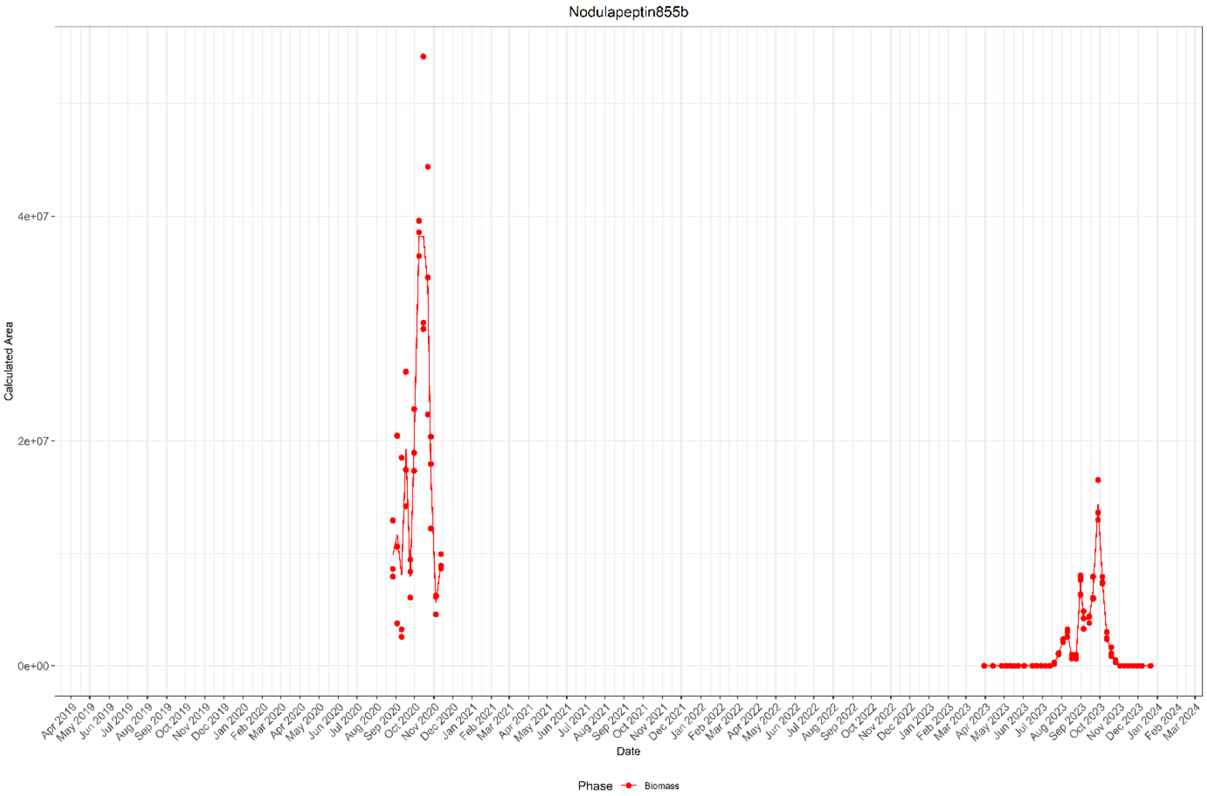

12. Ferintoic acid A

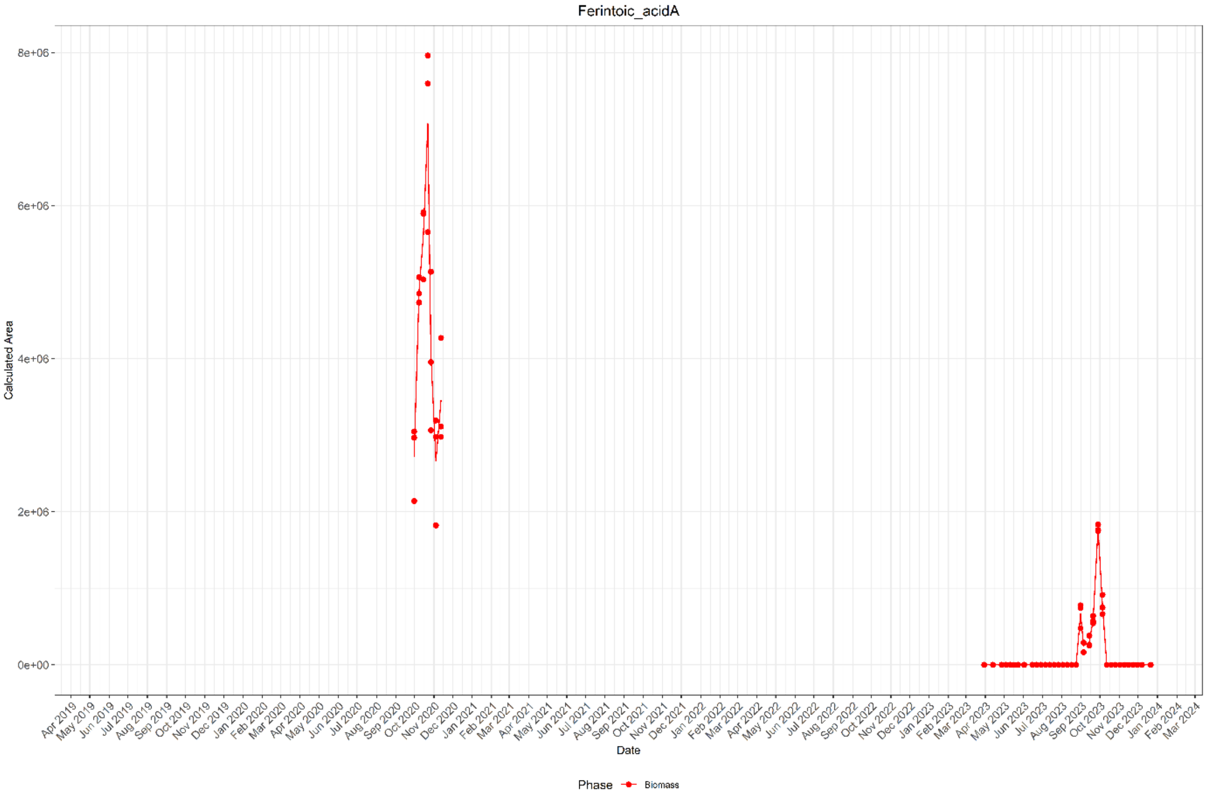

13. Ferintoic acid B

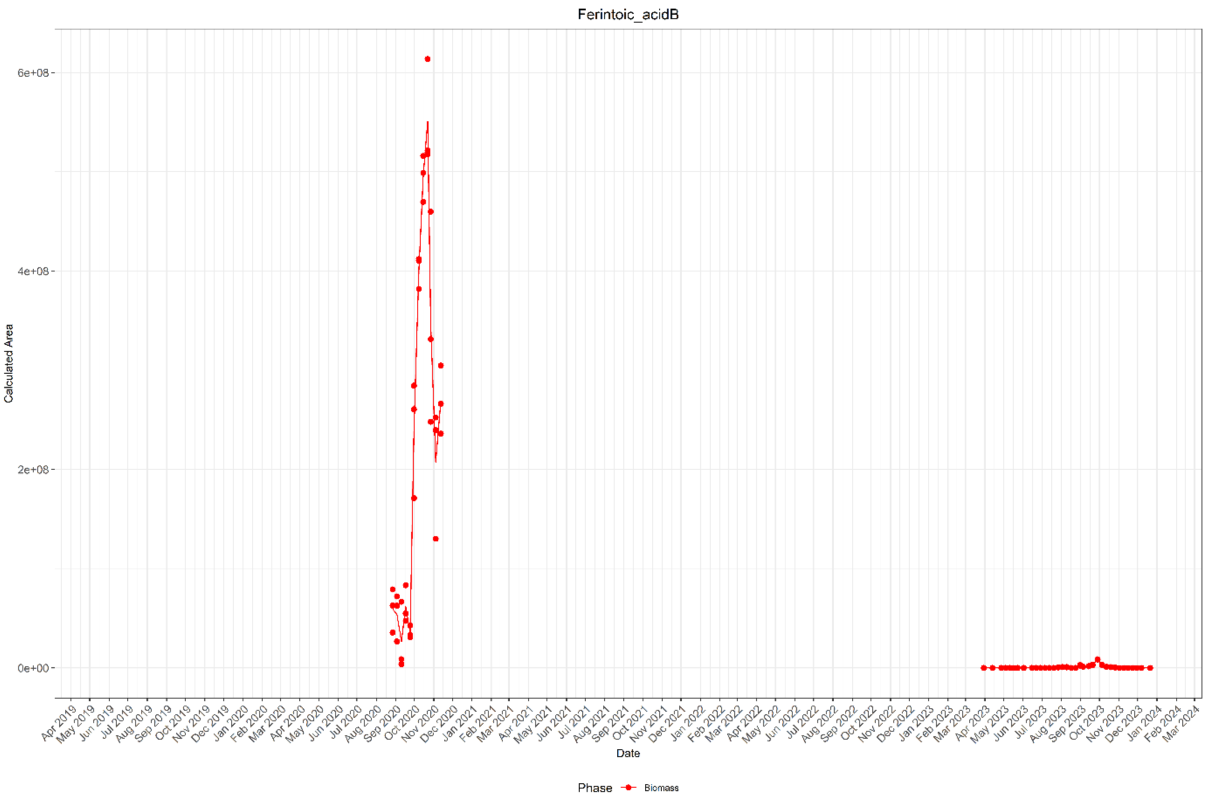

14. MC-KynA

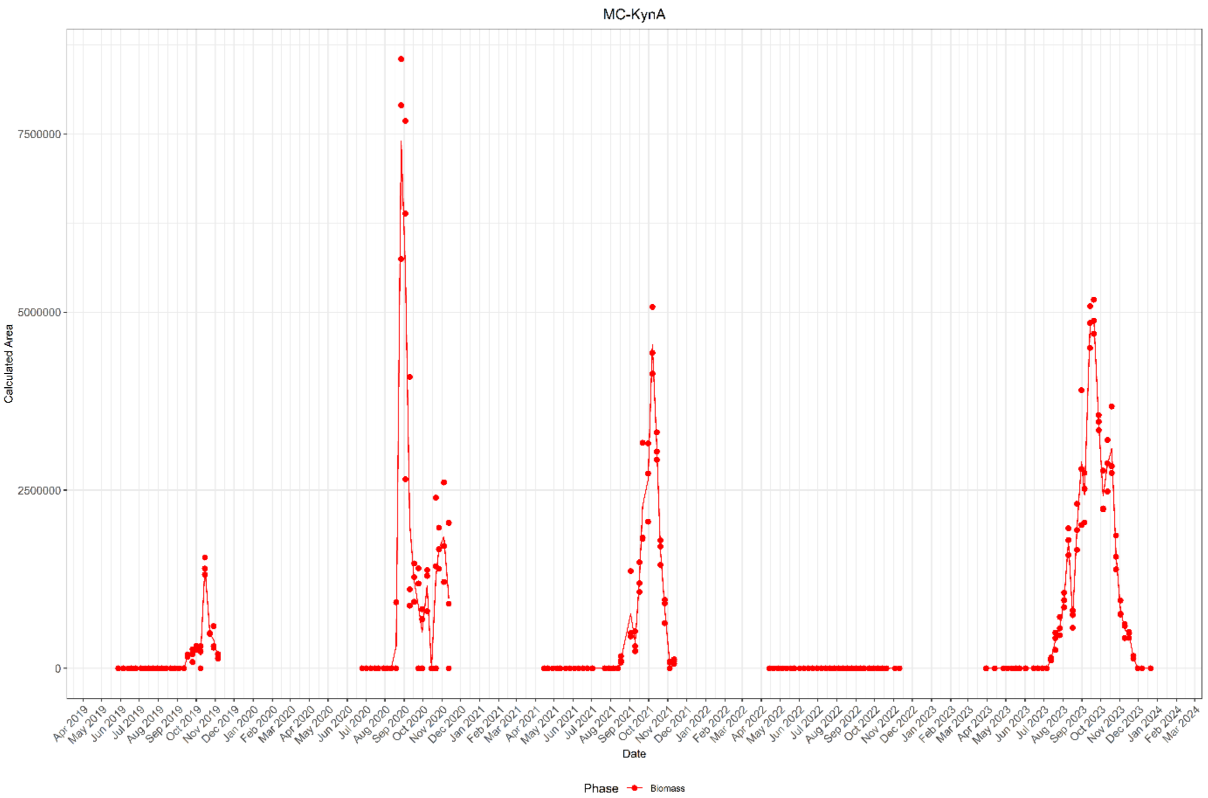

15. [NMe-Ala7] MC-LR

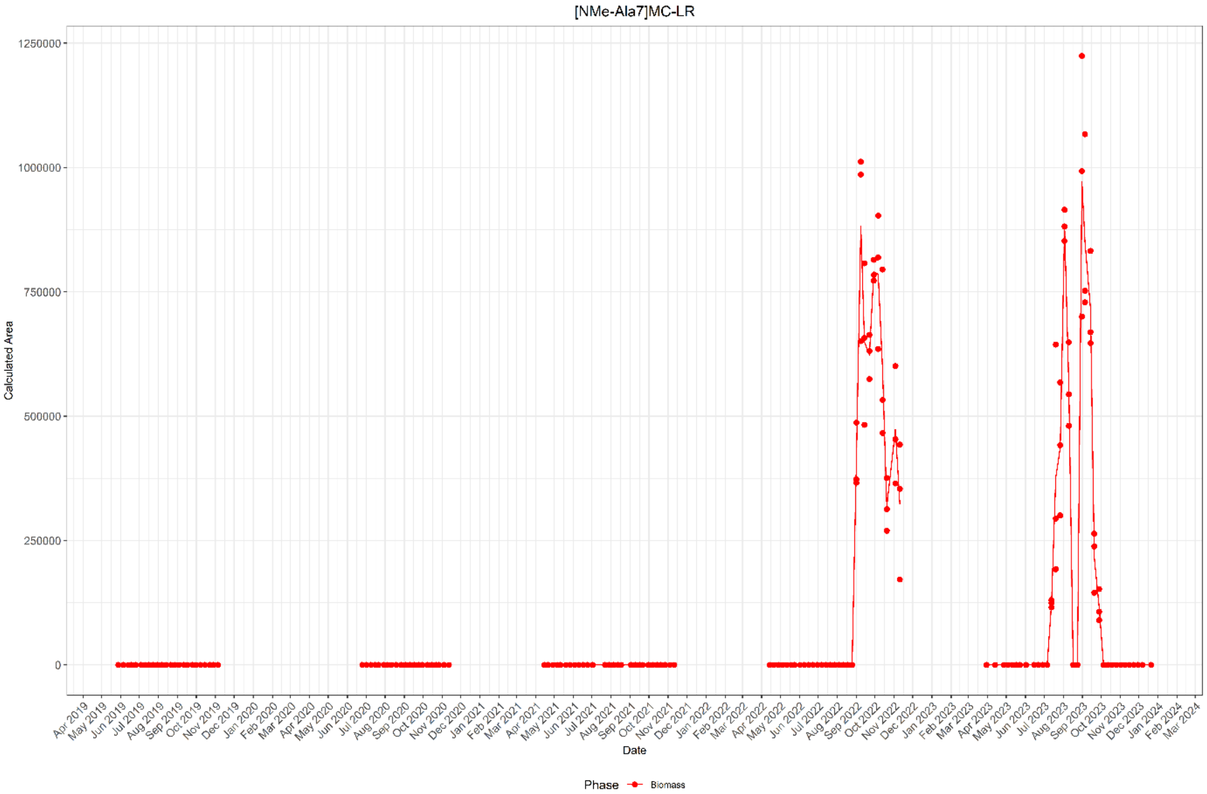

16. MC-(H2)YA

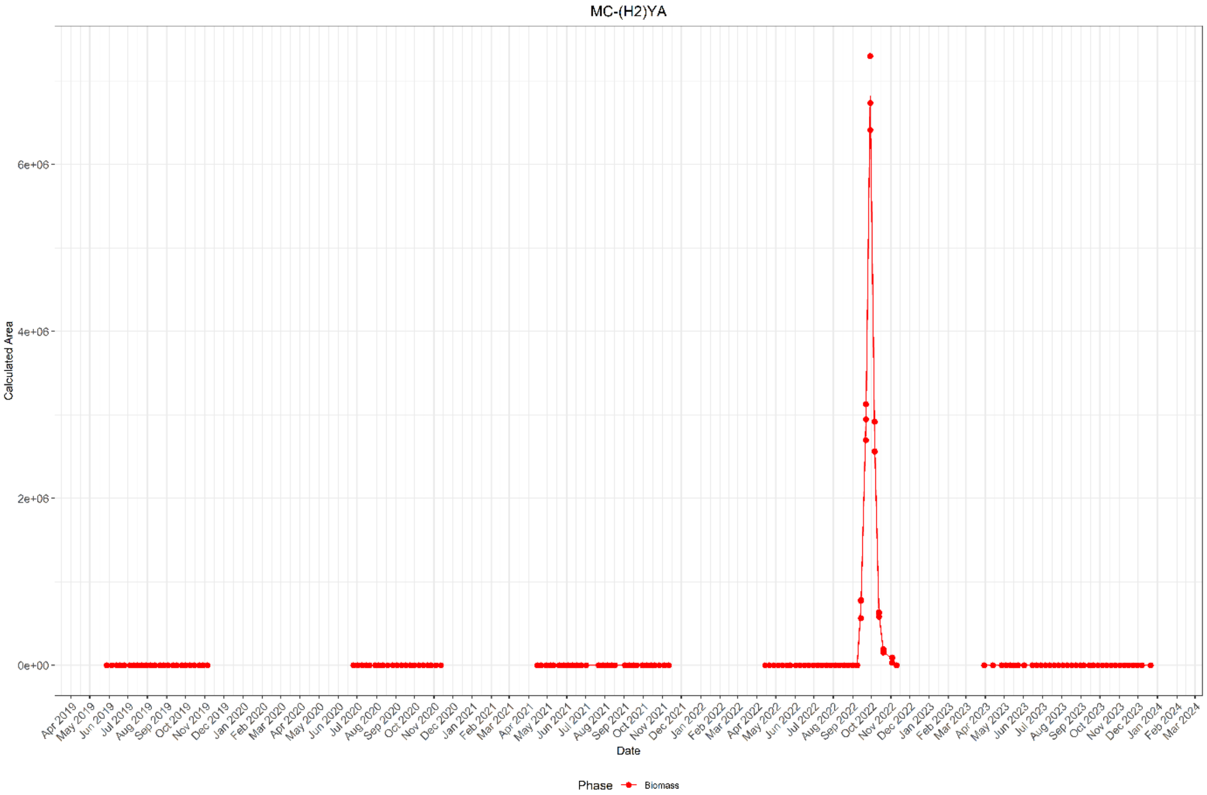

17. [D-Leu[1]] MC-HphR

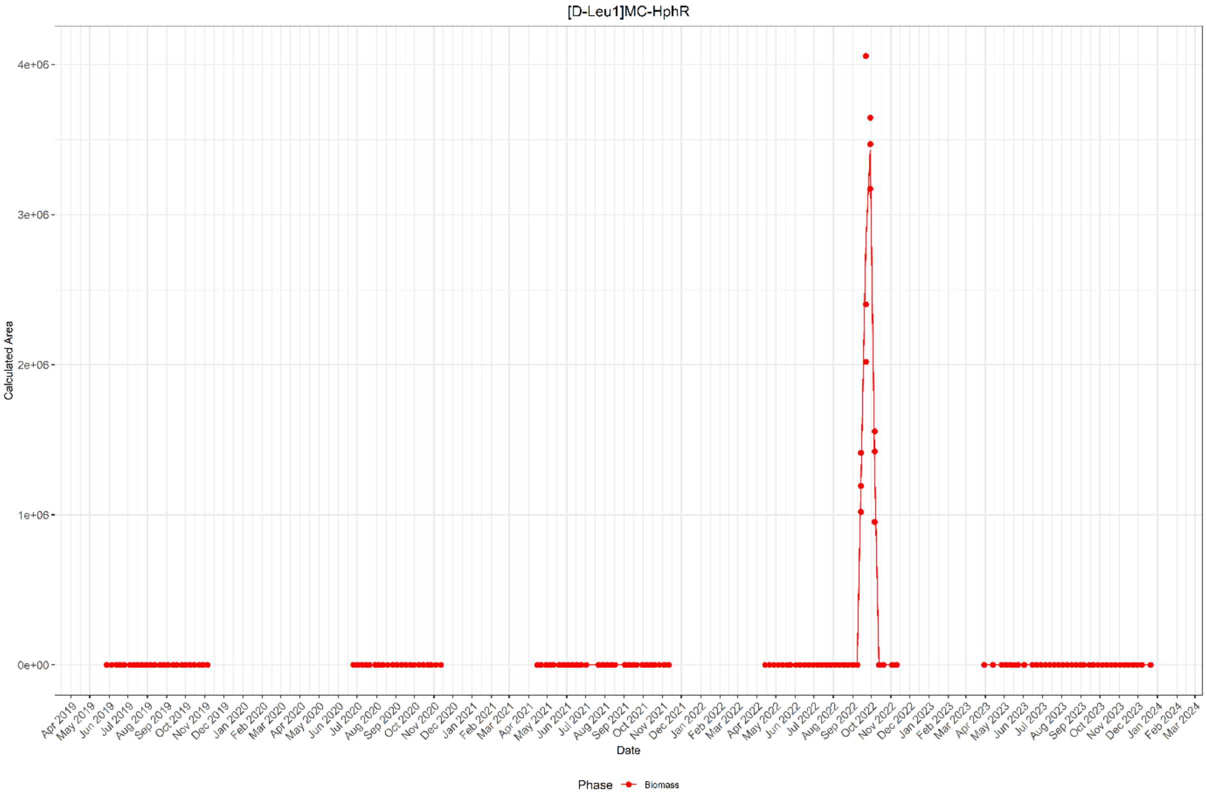

18. Microginin 580

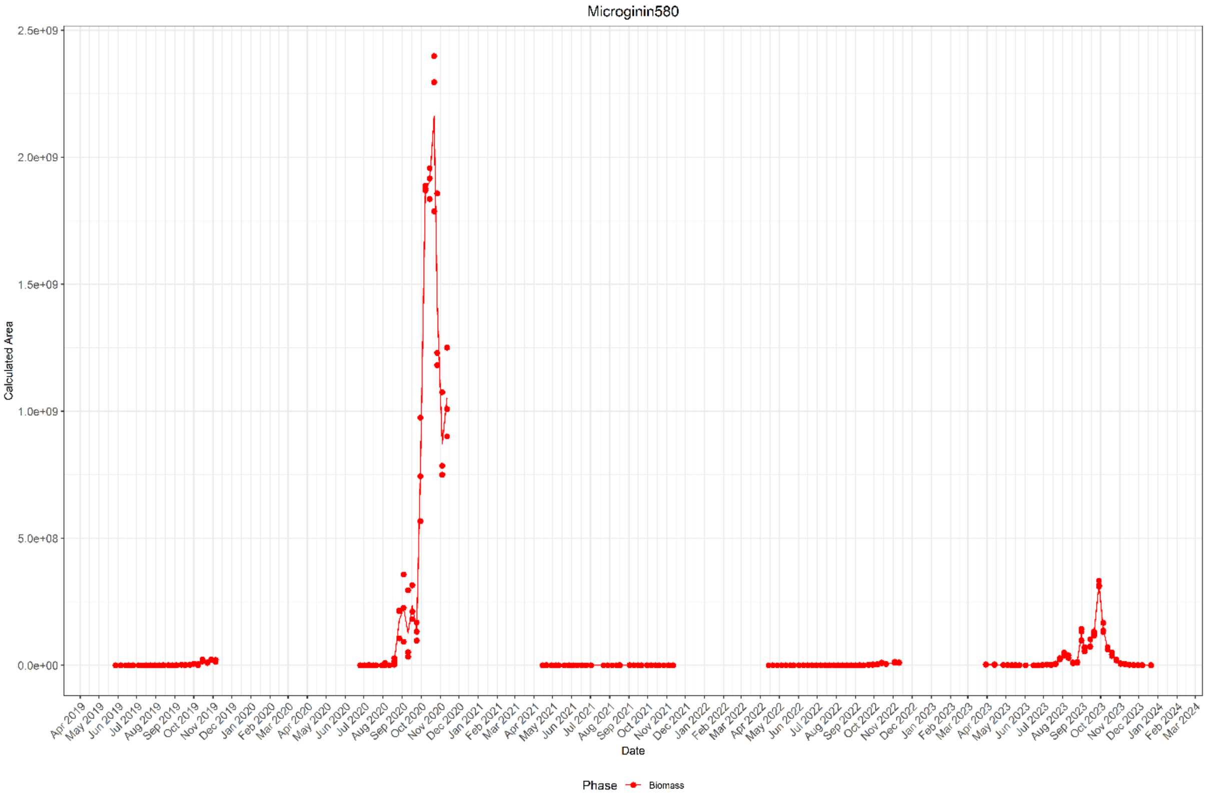

19. Microginin 757

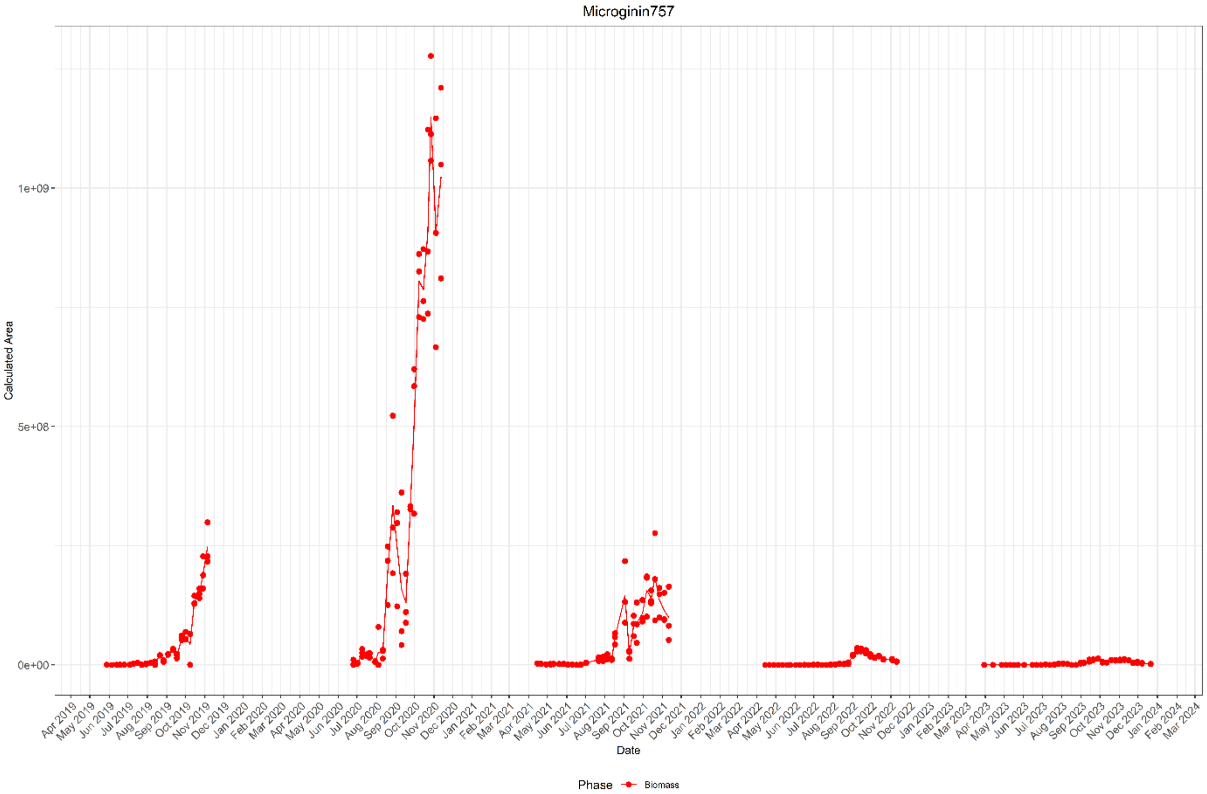

20. Microginin 791

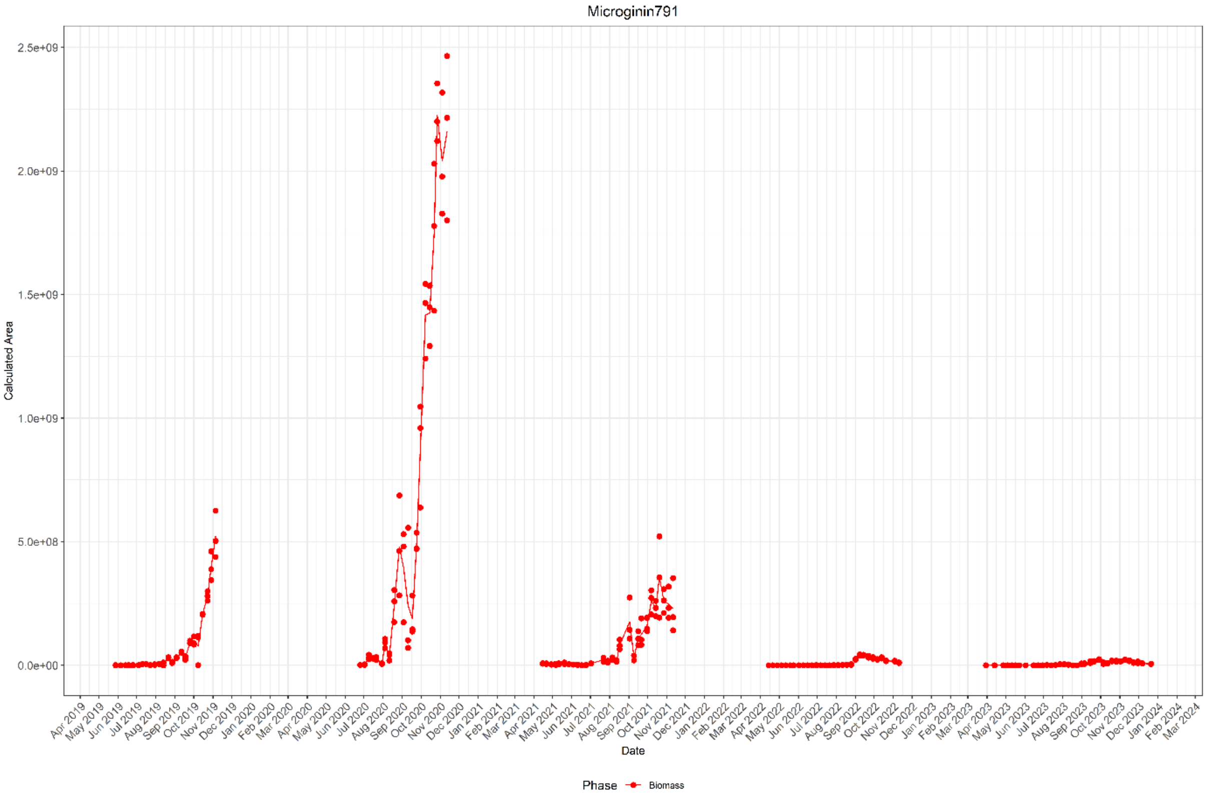

21. Microginin 761B

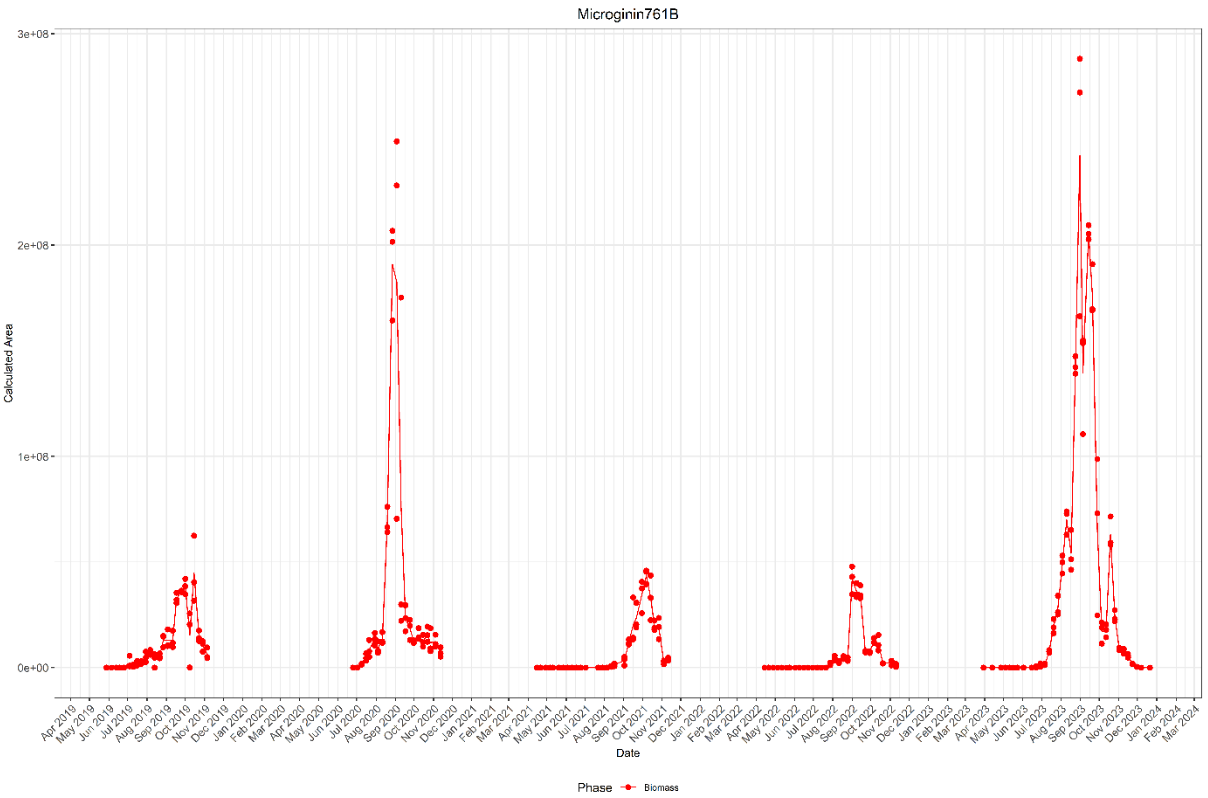

22. Microginin FR5

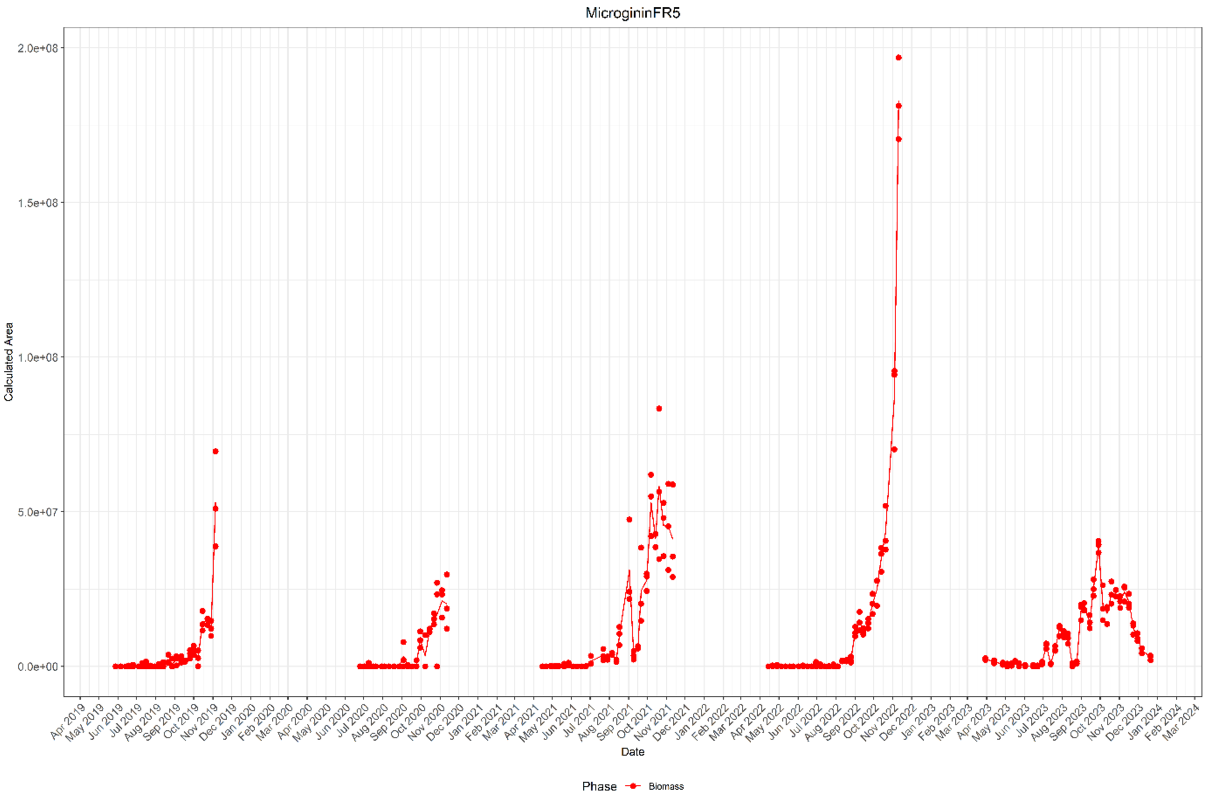

23. Oscillaginin A

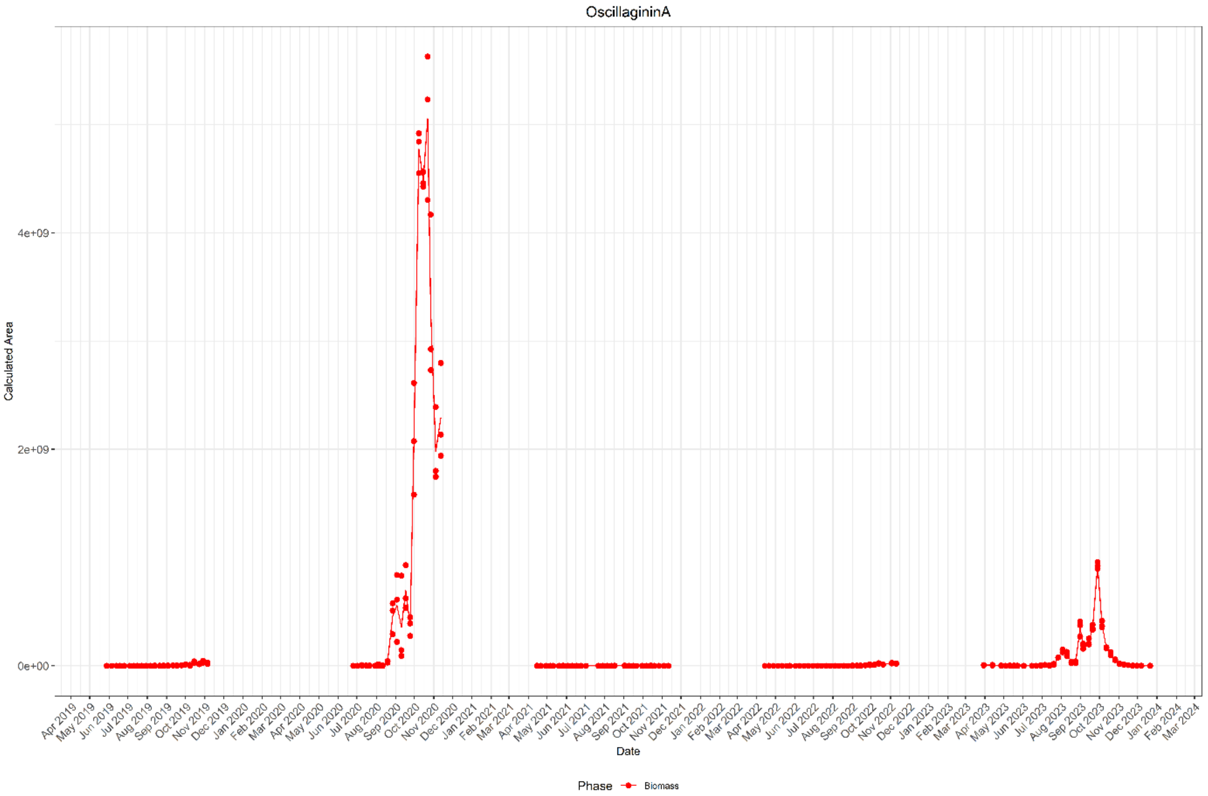

24. Aeruginosamide

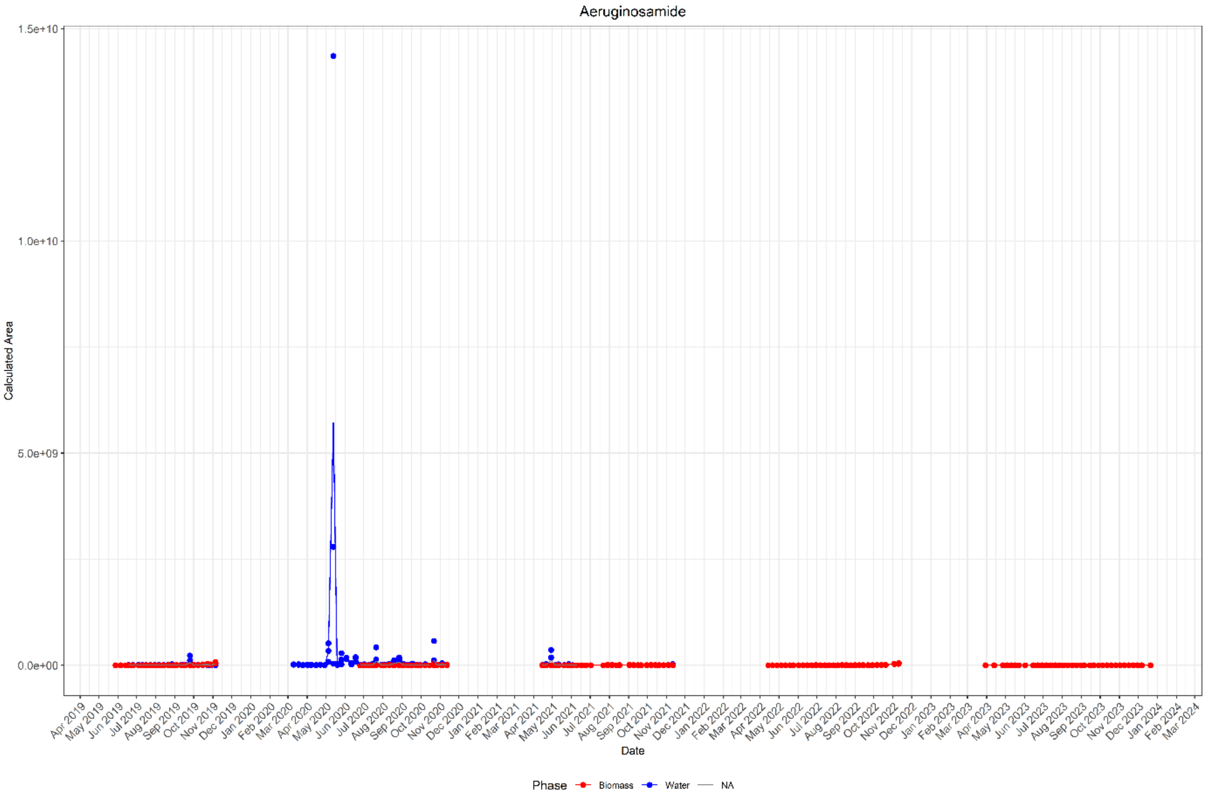

25. Planktocyclin

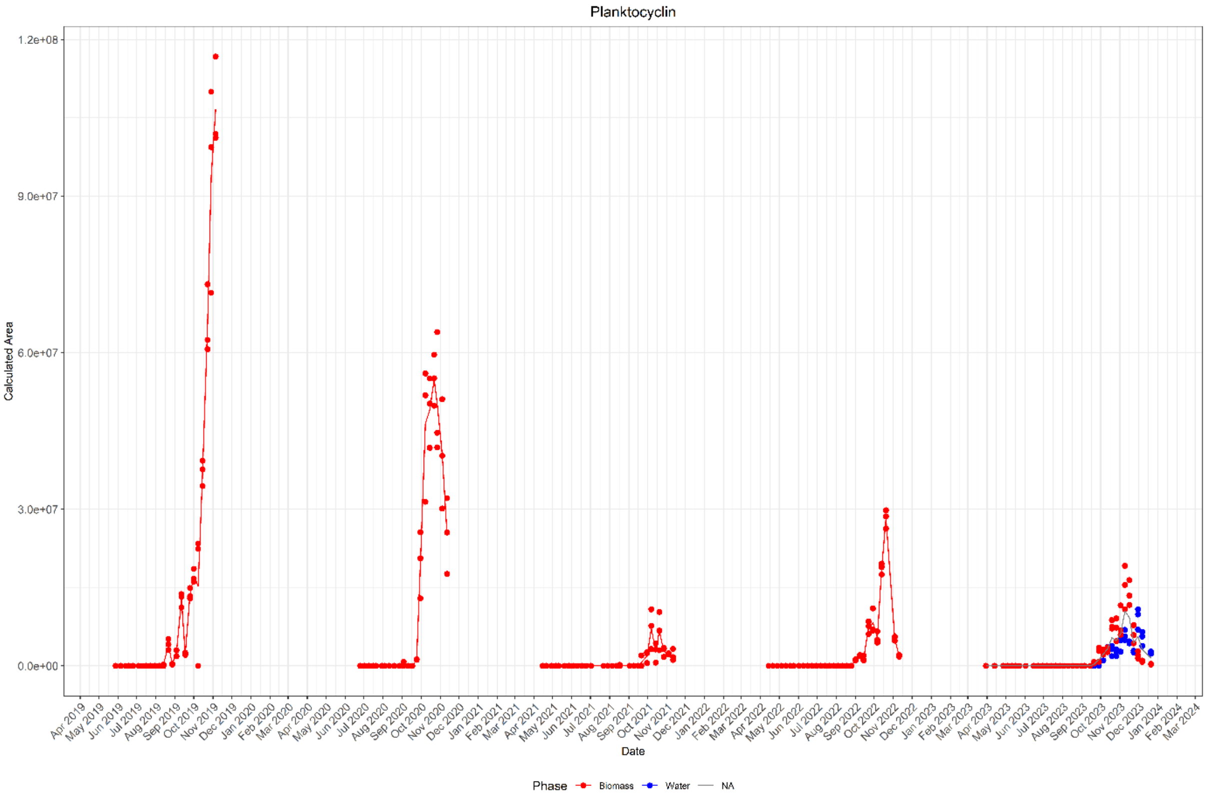

26. Planktopeptin BL1125

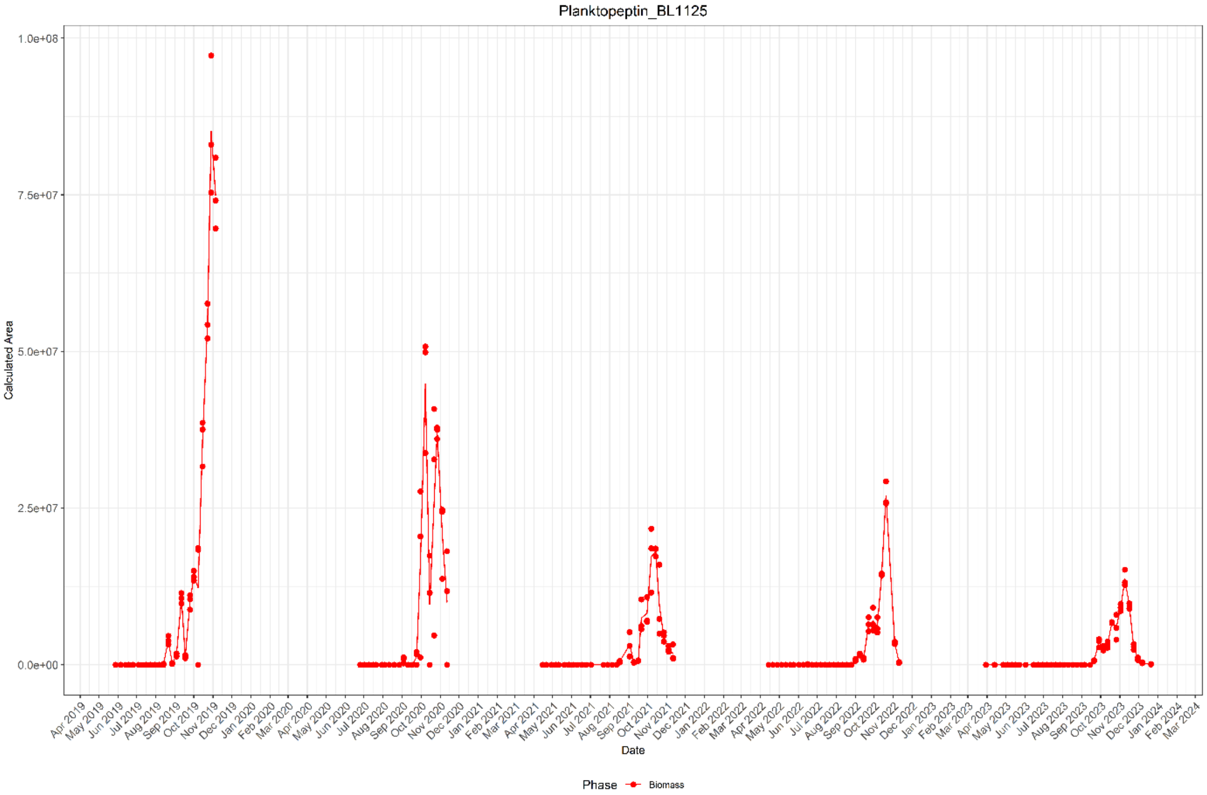

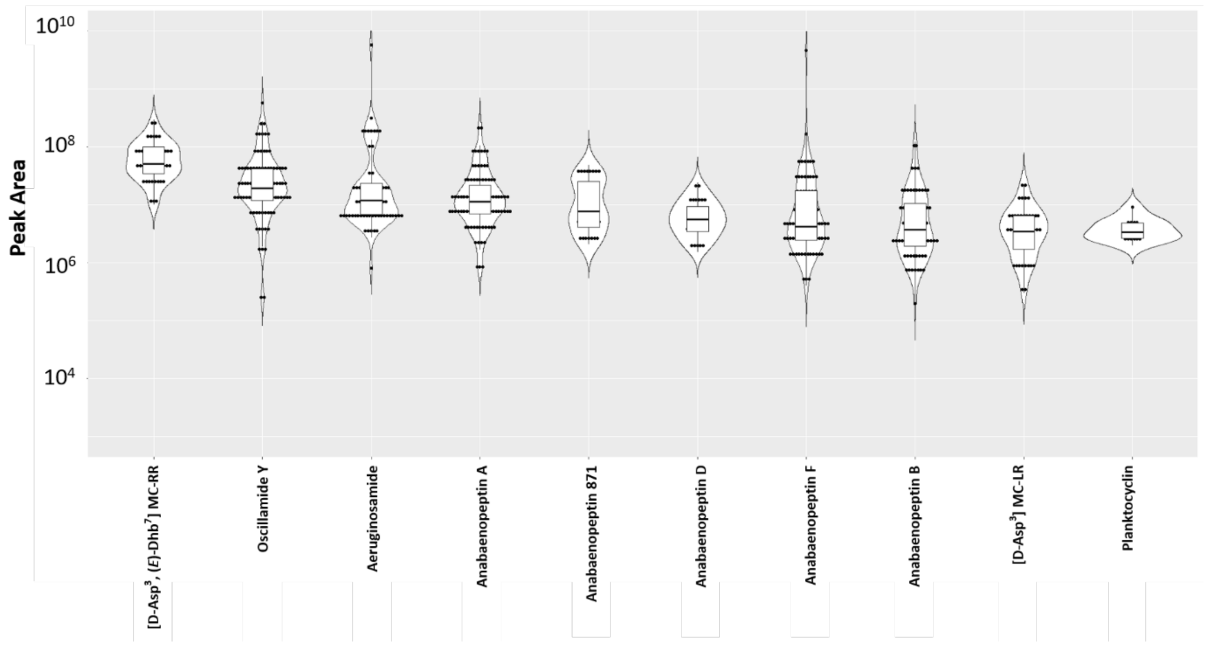

Figure S5. Violin plots showing LC-MS peak areas for cyanobacterial metabolites detected in aqueous samples across the 5-year sampling campaign (2019-2023) in Lake Greifensee. These compounds were annotated to confidence level 2, based on consideration of LC-MS and MS2 data.

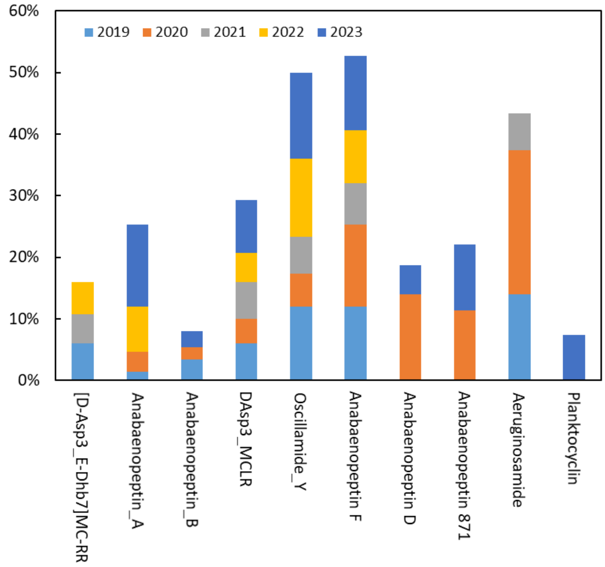

Figure S6. Detection frequency (%) of cyanobacterial metabolites in aqueous samples across the 5-year sampling campaign (2019-2023) in Lake Greifensee.

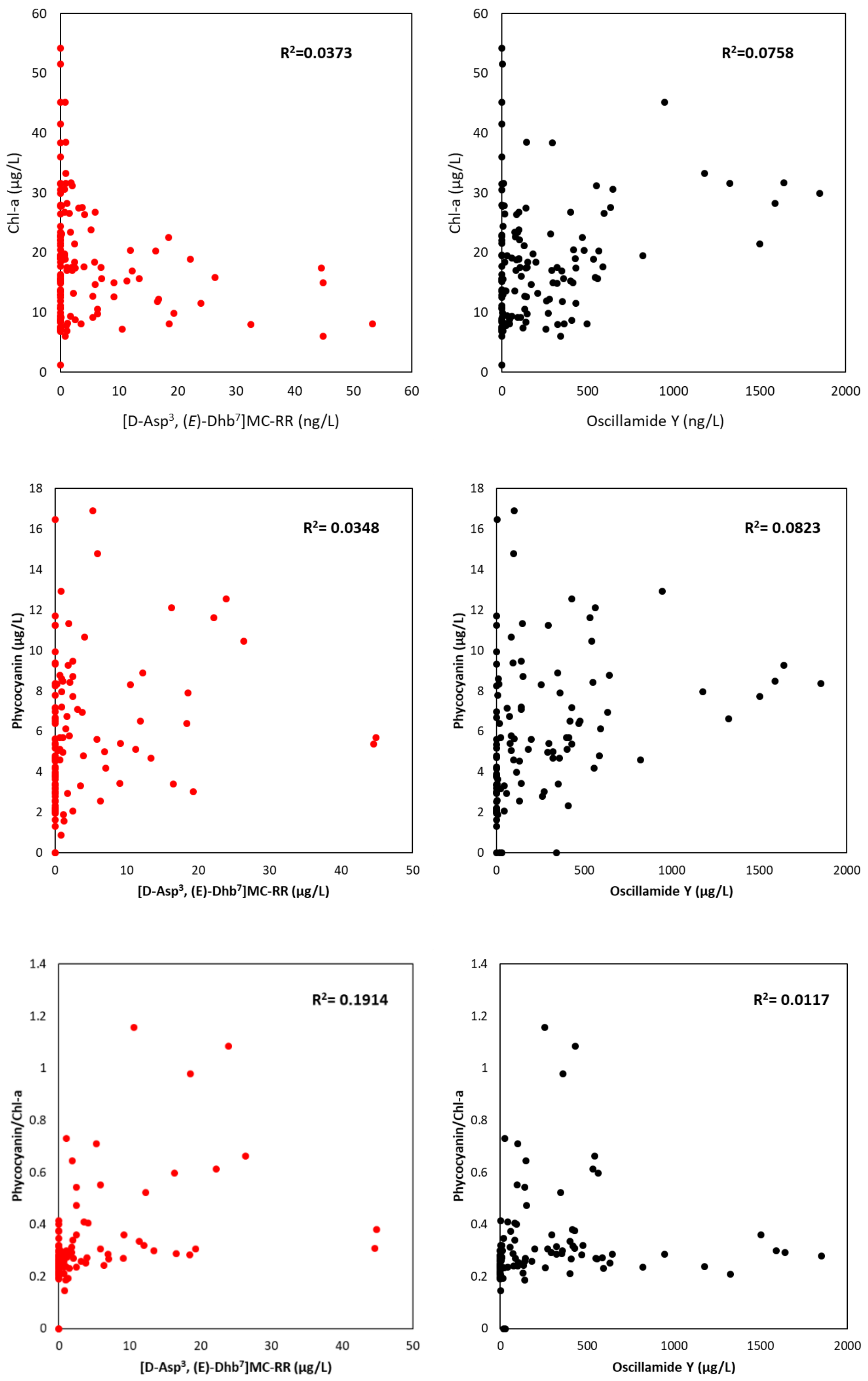

Figure S7. Co-variance of Chlorophyll-a (top), phycocyanin (middle), and the phycocyanin to Chl-a ratio (bottom) with [D-Asp^3, (*E*)-Dhb^7]MC-RR (left) and Oscillamide Y (right) concentrations with the respective linear correlation coefficients listed (r^2 values).

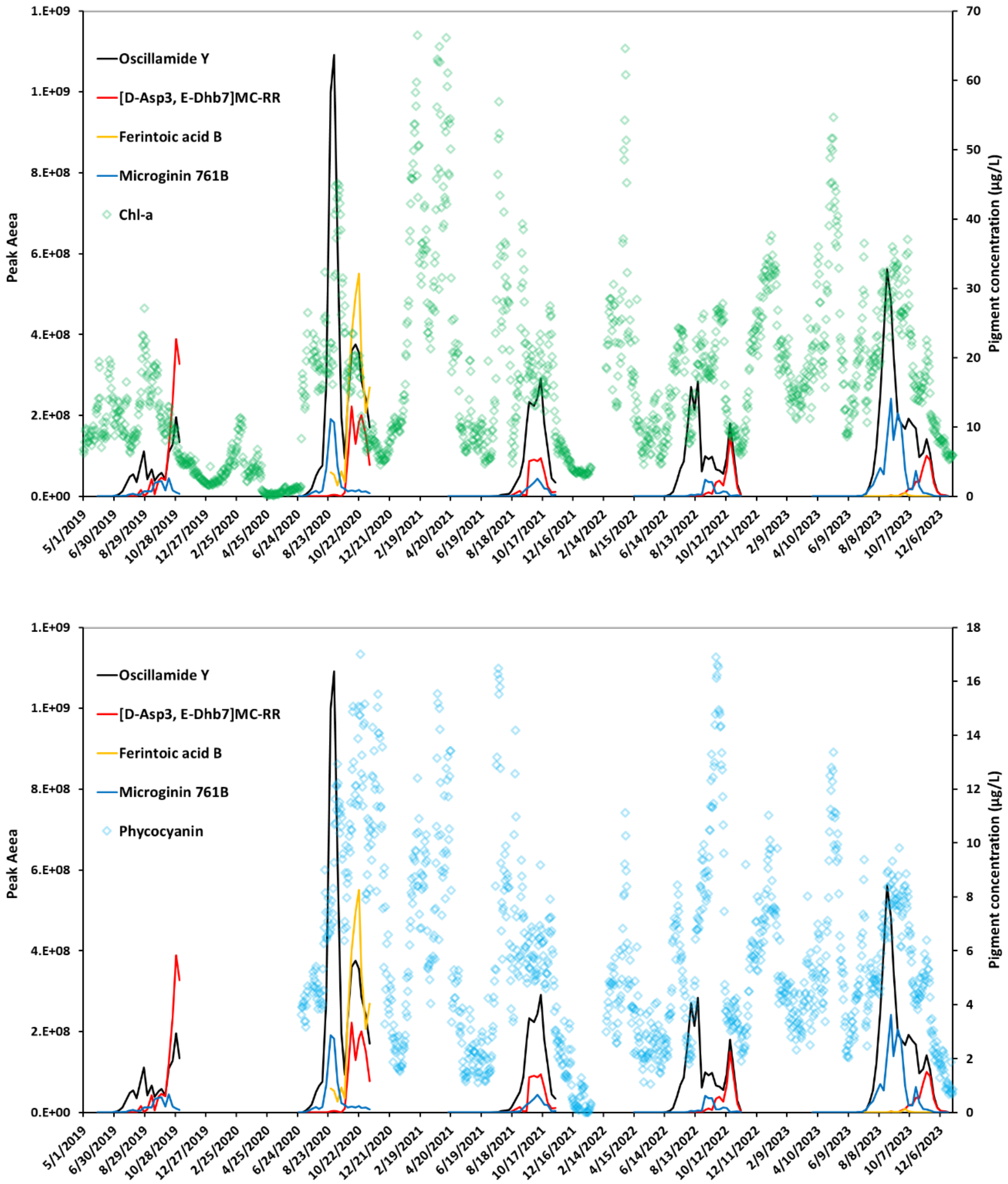

Figure S8. Time-series of indicator metabolites: Oscillamide Y (black) produced by all three species from Lake Greifensee, [D-Asp^3, (*E*)-Dhb^7]MC-RR (red) produced by *Planktothrix* G2020 isolate, Microginin 761B (blue) produced by *Microcystis* G2011 isolate, Ferintoic acid B (yellow ocher) produced by *Microcystis* G2020 isolate and variation of Chl-a (green, top) and phycocyanin (blue, bottom) on the secondary y-axis.

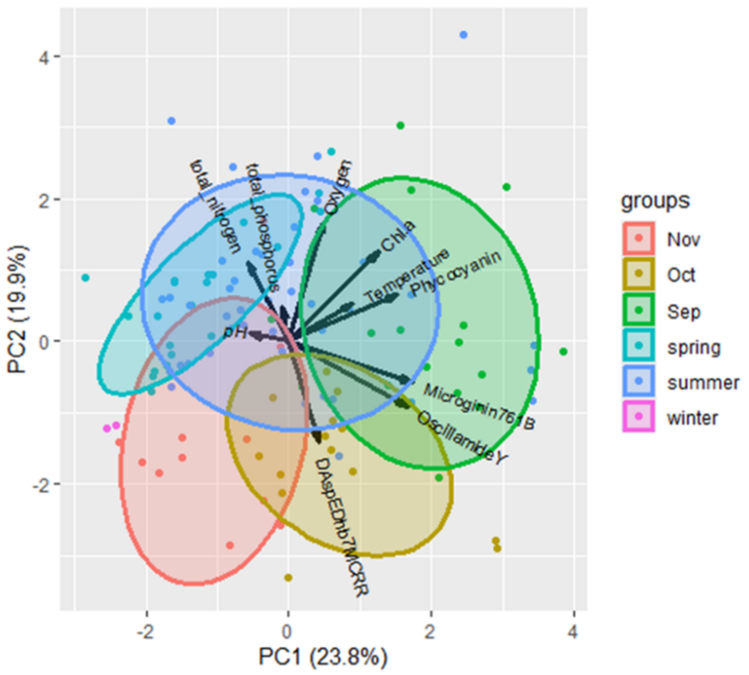

Figure S9. Principal component analysis including three cyanobacterial metabolites and monitoring parameters (pH, Chl-a, phycocyanin, water temperature, dissolved oxygen, total phosphorous and total nitrogen). Data used for the analysis was from July 2020 to December 2023 as no data was available for phycocyanin before July 2020. Time points with missing values were ignored for the respective parameter (“stats”, “ggplot2” and “ggbiplot” packages were used in R studio).

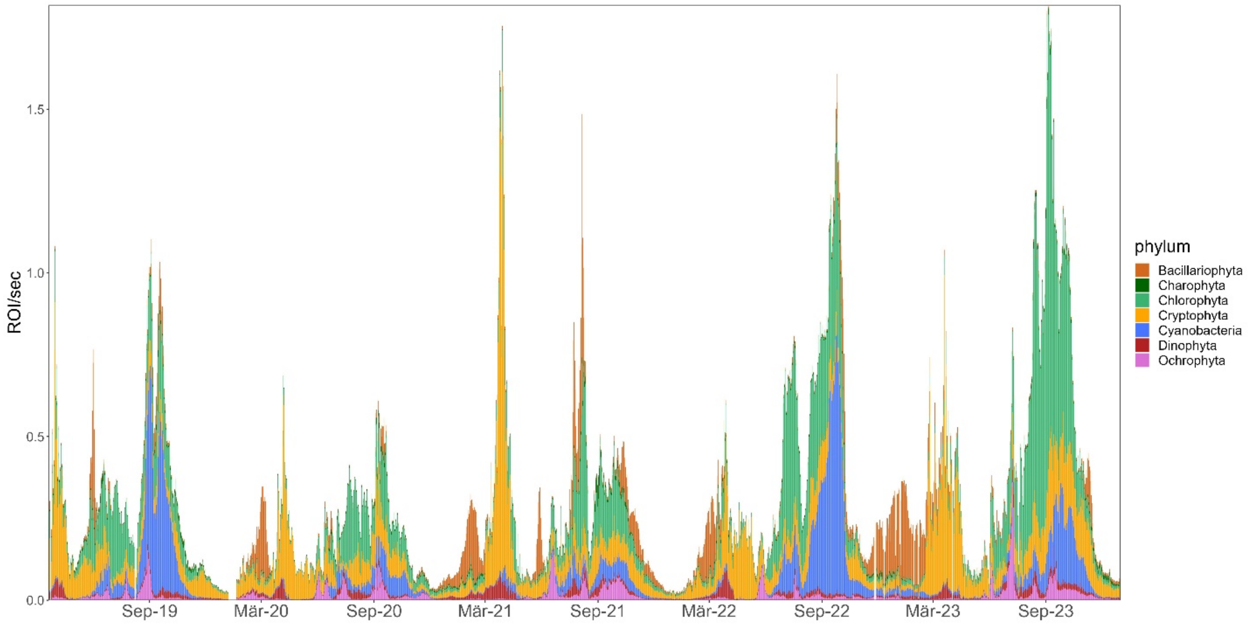

Figure S10. Time-series of phytoplankton abundance obtained by automated underwater imaging at 3 m in Lake Greifensee from 2019 to 2023. ROI = region of interest: this represents each identified plankton object, which was subsequently classified and aggregated at the phylum level. Abundances are expressed as ROI/s, as the imaging instrument captures objects per unit of time. For details about how plankton data were collected, classified and analysed see Merz et al. 2021.

Reference:

(Method: Merz, E., Kozakiewicz, T., Reyes, M., Ebi, C., Isles, P., Baity-Jesi, M., Roberts, P., Jaffe, J. S., Dennis, S. R., Hardeman, T., Stevens, N., Lorimer, T., & Pomati, F. (2021). Underwater dual-magnification imaging for automated lake plankton monitoring. Water Research, 203, 117524. https://doi.org/https://doi.org/10.1016/j.watres.2021.117524

Data are available at: https://doi.org/10.25678/000C2G)

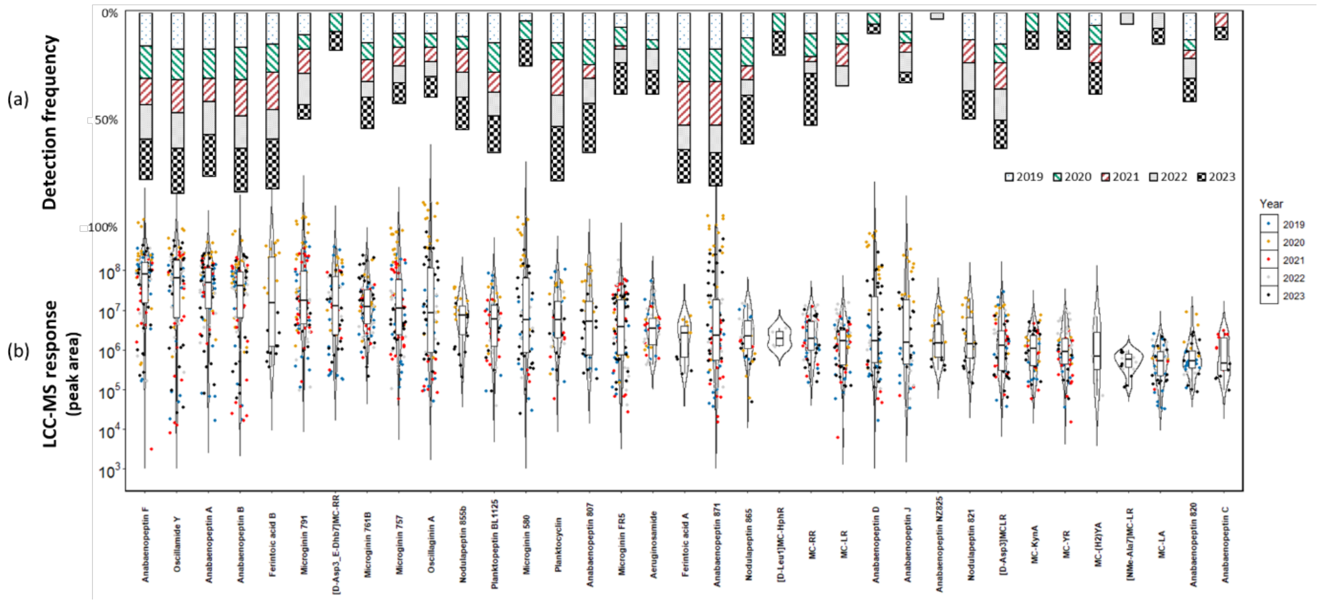

Figure S11. Analogous to Figure 2 in the main manuscript with colors changed to accommodate color-blind people. (a) Relative detection frequency and (b) LC-MS response (peak area) for all 26 metabolites identified in Lake Greifensee over a 5-year sampling campaign spanning from 2019 to 2023.

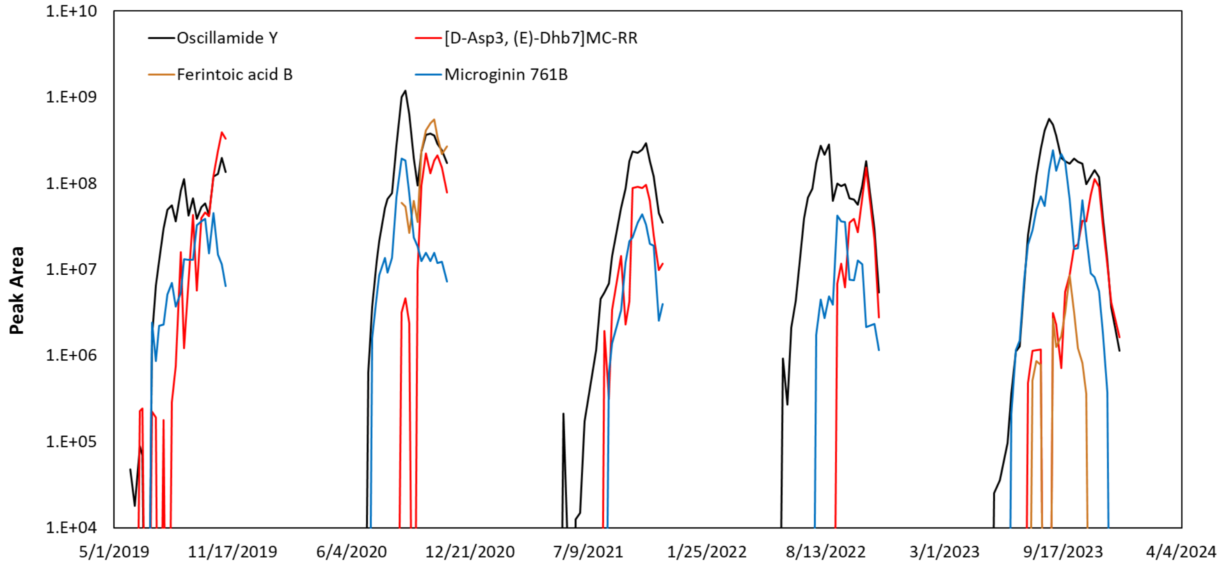

Figure S12. Time-series of indicator metabolites: Oscillamide Y (black) produced by all three species from Lake Greifensee, [D-Asp^3, (*E*)-Dhb^7]MC-RR (red) produced by *Planktothrix* G2020 isolate, Microginin 761B (blue) produced by *Microcystis* G2011 isolate, Ferintoic acid B (yellow ocher) produced by *Microcystis* G2020 isolate (identical to Figure 3 in main manuscript but with y-axis in log-scale).
